# Increased Hemodynamic Load in Early Embryonic Stages Alters Endocardial to Mesenchymal Transition

**DOI:** 10.3389/fphys.2017.00056

**Published:** 2017-02-08

**Authors:** Madeline Midgett, Claudia S. López, Larry David, Alina Maloyan, Sandra Rugonyi

**Affiliations:** ^1^Biomedical Engineering, Oregon Health and Science UniversityPortland, OR, USA; ^2^Multiscale Microscopy Core, OHSU Center for Spatial Systems Biomedicine, Oregon Health and Science UniversityPortland, OR, USA; ^3^Proteomics Core, Oregon Health and Science UniversityPortland, OR, USA; ^4^Knight Cardiovascular Institute, Oregon Health and Science UniversityPortland, OR, USA

**Keywords:** hemodynamics, endothelial–mesenchymal transition, EMT, chick embryo, heart development, cardiac cushion

## Abstract

Normal blood flow is essential for proper heart formation during embryonic development, as abnormal hemodynamic load (blood pressure and shear stress) results in cardiac defects seen in congenital heart disease. However, the progressive detrimental remodeling processes that relate altered blood flow to cardiac defects remain unclear. Endothelial–mesenchymal cell transition is one of the many complex developmental events involved in transforming the early embryonic outflow tract into the aorta, pulmonary trunk, interventricular septum, and semilunar valves. This study elucidated the effects of increased hemodynamic load on endothelial–mesenchymal transition remodeling of the outflow tract cushions *in vivo*. Outflow tract banding was used to increase hemodynamic load in the chicken embryo heart between Hamburger and Hamilton stages 18 and 24. Increased hemodynamic load induced increased cell density in outflow tract cushions, fewer cells along the endocardial lining, endocardium junction disruption, and altered periostin expression as measured by confocal microscopy analysis. In addition, 3D focused ion beam scanning electron microscopy analysis determined that a portion of endocardial cells adopted a migratory shape after outflow tract banding that is more irregular, elongated, and with extensive cellular projections compared to normal cells. Proteomic mass-spectrometry analysis quantified altered protein composition after banding that is consistent with a more active stage of endothelial–mesenchymal transition. Outflow tract banding enhances the endothelial–mesenchymal transition phenotype during formation of the outflow tract cushions, suggesting that endothelial–mesenchymal transition is a critical developmental process that when disturbed by altered blood flow gives rise to cardiac malformation and defects.

## Introduction

Congenital heart defects affect nearly 1% of newborns and are the leading cause of infant death in the United States (Hoffman and Kaplan, 2002; Yang et al., 2006). Additionally, surgical repair of congenital heart defects is associated with a yearly economic burden of approximately $2.2 billion (Russo and Elixhauser, 2006) and long-term consequences for patients. Blood flow dynamics play a critical role in regulating early heart development (Culver and Dickinson, 2010), as numerous studies have shown that surgically altered blood flow results in a spectrum of cardiac defects seen in human congenital heart disease (CHD) (Clark and Rosenquist, 1978; Clark et al., 1989; Hogers et al., 1997, 1999; Sedmera et al., 1999; Tobita et al., 2002; Hu et al., 2009; Midgett and Rugonyi, 2014). However, the ways in which altered blood flow triggers malformation that leads to cardiac defects remain unclear. Understanding the root causes of CHD and the initial dysregulation leading to it is essential for future prevention and treatment.

The heart is the first functional organ in the embryo, and starts beating and pumping blood as soon as the primitive heart tube is formed. Blood is ejected from the ventricle of the tubular heart into the arterial vessel system via the outflow tract. Early during cardiac development, endocardial cushions (localized thickenings of the wall) form in the outflow tract and atrioventricular canal, and eventually serve as primitive valves that block blood flow upon contraction of the myocardium. The endocardial cushions are initially composed of extracellular matrix (cardiac jelly), separated from blood by an endocardium monolayer, and collectively adapt to an ever-changing biomechanical environment as blood pressure and flow increase over development. The outflow tract cushions are frequently studied because they are very sensitive to hemodynamic perturbation and later transform into the interventricular septum and semilunar valves, which are often involved in congenital heart defects (Hogers et al., 1997; Hove et al., 2003; Gittenberger-de Groot et al., 2005).

Endothelial mesenchymal transition (EMT) (Runyan et al., 2000) is considered a hallmark process in the development of the endocardial cushions. EMT characterizes a complex process in endocardial cushion formation, where a portion of endocardial cells delaminate from the endocardium monolayer, invade the cardiac jelly, and then proliferate and differentiate into mesenchymal cells, leaving the disassembled endocardium to rearrange, and form a new intact flattened layer (Icardo, 1989; Person et al., 2005). EMT in the outflow tract cushions ultimately leads to formation of the semilunar valves, and is regulated by intricate signaling pathways that include TGFβ, Notch, and Erbb3 (Lencinas et al., 2011; Lim and Thiery, 2012). These EMT pathways depend on many extracellular matrix components, soluble growth factors, and various transcription factors that promote a mesenchymal phenotype by stimulating the disassembly of cell junction complexes and re-arrangement of actin cytoskeleton during EMT activation, as well as re-organization of extracellular matrix during cell invasion. While EMT is still not completely understood, *in vitro* culture systems, *ex vivo* cushion explant systems, and knockout mouse models have defined key factors governing EMT in the outflow tract and atrioventricular cushions (Armstrong and Bischoff, 2004) and identified several EMT markers (Zeisberg and Neilson, 2009). Both sets of endocardial cushions follow similar developmental mechanisms, however EMT in the outflow tract cushions lag behind and cushion development has an additional cellular contribution from neural crest cells in the cardiac jelly by HH21 (Webb et al., 2003; Hinton and Yutzey, 2011). Cushion explant studies have revealed an important role of soluble growth factors within the extracellular matrix (Eisenberg and Markwald, 1995) and a unique cushion response to critical myocardial-derived differentiation signals (Runyan and Markwald, 1983), while mouse models have elucidated dozens of EMT signaling gene disruptions that alter valve phenotypes (Gitler et al., 2003; Schroeder et al., 2003). In addition, several studies also indicated that mechanotransduction signaling is key to normal cushion development, where increased shear stress activates TGFβ-dependent Krüppel like factor 2 (KLF2) signaling in endothelial cells *in vitro* (Egorova et al., 2011a,b), and contractile mechanical forces modulate EMT *ex vivo* (Sewell-Loftin et al., 2014). Intriguingly misregulation of signaling pathways (Hinton et al., 2006) and hemodynamic surgical interventions (Midgett and Rugonyi, 2014) lead to similar cardiac deficits. However, the modulating effects of blood flow on EMT have not been fully elucidated.

This study investigated the effects of increased hemodynamic load (blood pressure and wall shear stress) on outflow tract cushion EMT in early development. Hemodynamic forces exerted by blood flow on heart tissue walls trigger mechanotransduction mechanisms that lead to physical, chemical, and gene regulatory responses in cardiac tissue (Davies, 1995). To alter blood flow through the heart, this study used a well-established hemodynamic intervention called outflow tract banding in the chicken embryo at Hamburger and Hamilton (HH) stage 18 (~3 days of incubation; Hamburger and Hamilton, 1992). Outflow tract banding increases peak ventricular pressure (Tobita et al., 2002; Shi et al., 2013) and blood flow velocities (Rugonyi et al., 2008; Midgett et al., 2014) in the outflow cushion region. These hemodynamic changes are dependent on the degree of band tightness (Midgett et al., 2014) and result in a wide spectrum of heart defects in the chicken embryo (Clark and Rosenquist, 1978; Clark et al., 1989; Hogers et al., 1997; Sedmera et al., 1999; Tobita et al., 2002). We used chicken embryos as a model of human heart development (which is highly conserved among vertebrate species) to allow for ease of accessibility in the egg for surgical manipulation and *in vivo* imaging (McQuinn et al., 2007; Rugonyi et al., 2008; Shi et al., 2013). Banding was performed at the onset of EMT (Person et al., 2005) in the outflow tract cushions in order to characterize changes in normal cushion development induced by increased hemodynamic load *in vivo*. Changes in shape and organization of cellular and extracellular matrix components of outflow tract tissue after banding were characterized with immunohistochemistry, confocal microscopy, 3D electron microscopy, and combined with proteomics analysis to characterize the ways in which normal tissue remodeling is detrimentally modified by increased hemodynamic load. Specifically, endocardial and cardiac jelly cell density and organization were quantified with DAPI and phalloidin staining, VE-cadherin endocardial junctions and extracellular matrix periostin organization (markers of EMT) were analyzed with immunolabeling, 3D endocardial cell arrangements were quantified with electron microscopy, and global changes in relative protein abundances were measured by mass spectrometry.

## Materials and methods

### Hemodynamic intervention

Fertilized White Leghorn chicken eggs were incubated blunt end up at 38°C and 80% humidity until stage HH18 (~3 days; Hamburger and Hamilton, 1992). Chick embryos are not considered vertebrates under IACUC and Oregon Health & Science University regulations, however we made every effort to minimize the number of embryos needed. Embryo hemodynamics were altered with a 10-0 nylon suture passed under the outflow tract and tied in a knot around the mid-section of the outflow tract to constrict the cross-sectional area. A control group of embryos served as a surgical sham where the suture was passed under the outflow tract but not tightened. Following interventions, eggs were sealed with saran wrap and incubated until further evaluation.

### Band tightness measurement with OCT

A custom-made optical coherence tomography (OCT) system was used to measure chick embryo band tightness *in vivo* as previously described (Rugonyi et al., 2008; Ma et al., 2010; Liu et al., 2012; Shi et al., 2013). Briefly, the system has a spectral domain configuration with a super luminescent diode centered at 1325 nm from Thorlabs Inc. (Newton, NJ, USA) and a 1024 pixel, 92 kHz maximal line-scan rate infrared InGaAs line-scan camera from Goodrich Inc. (Charlotte, NC, USA). It acquired 512 × 512 pixel, 2D B-mode line-scan tomographic images at 140 frames per second with <10 μm resolution. Embryo temperature during acquisition was maintained at a normal physiological range (~38°C) with a thermocouple-controlled heating system. Each banded embryo was imaged immediately before and 2 h after manipulation with OCT to acquire 200 tomographic frames (~3–4 cardiac cycles) of the longitudinal outflow tract in order to measure the change in outflow tract diameter and calculate the degree of band tightness with Equation (1),

(1) $$\text{Band tightness}=1-{\text{D}}_{\text{a}}/{\text{D}}_{\text{b}},$$

where D_a_ is the maximum external diameter of the outflow tract at the band site after banding, and D_b_ is the maximum external diameter of the outflow tract at the approximate band site location before banding. The measured band tightness of each banded embryo was used to define the hemodynamic environment, based on our previous characterization of the relationship between the degree of outflow tract band tightness and the specific blood pressure and velocity conditions induced (Shi et al., 2013; Midgett et al., 2014). Band tightness in this study ranged from 0 to 60% constriction. After OCT imaging, the eggs were re-sealed with saran wrap and placed back in the incubator.

### Confocal microscopy

Immunohistochemistry and confocal microscopy were performed to characterize cushion remodeling after banding flow conditions. Whole embryos were removed from the egg at HH24 (~24 h after surgical manipulation at HH18) and fixed overnight in 4% paraformaldehyde at 4°C and dehydrated in methanol. Samples were then transferred into methanol/DMSO/H_2_O_2_ to block endogenous peroxidase activity, rehydrated, blocked for 45 min with PBS +10% TritonX +2% milk +4% goat sera, and treated with conjugated antibody (Alexa Fluor® 555 Antibody Labeling Kit, Life Technologies™) overnight at 4°C. Immunohistochemistry was performed with 1:100 rabbit anti-human VE-Cadherin (abcam®, ab33168) to label endothelial cell-cell adhesions (Runyan et al., 2000), and separately with 1:100 rabbit anti-mouse periostin (Aviva Systems Biology, OAPC00052) to label periostin fibers, which serve as a critical TGFβ signal transduction regulator and an EMT-stimulating scaffold for cell attachment and migration through the cardiac jelly (Nakajima et al., 2000; Kern et al., 2005). Separate embryos were stained to label F-actin with 66 nM Alexa Fluor® 568 Phalloidin (Molecular Probes™, A12380) in order to analyze cell organization within the outflow tract cushions. Normal (without intervention) embryo subsets were also stained with phalloidin and periostin between the stages of HH15 and HH25 in the outflow tract and the atrioventricular cushions to track F-actin and periostin remodeling changes during normal EMT progression. All samples were then extensively washed and co-stained with 10 nM DAPI (Life Technologies™, D1306) for 12 h at 4°C, before methanol dehydration and treatment with a clearing agent (1:2 benzyl alcohol: benzyl benzoate) prior to imaging. Confocal microscopy was performed with a Zeiss LSM 780 confocal laser scanning microscope (Carl Zeiss), and images were collected in a z-stack throughout the outflow cushions, spaced 10 μm apart.

**Figure 1:**
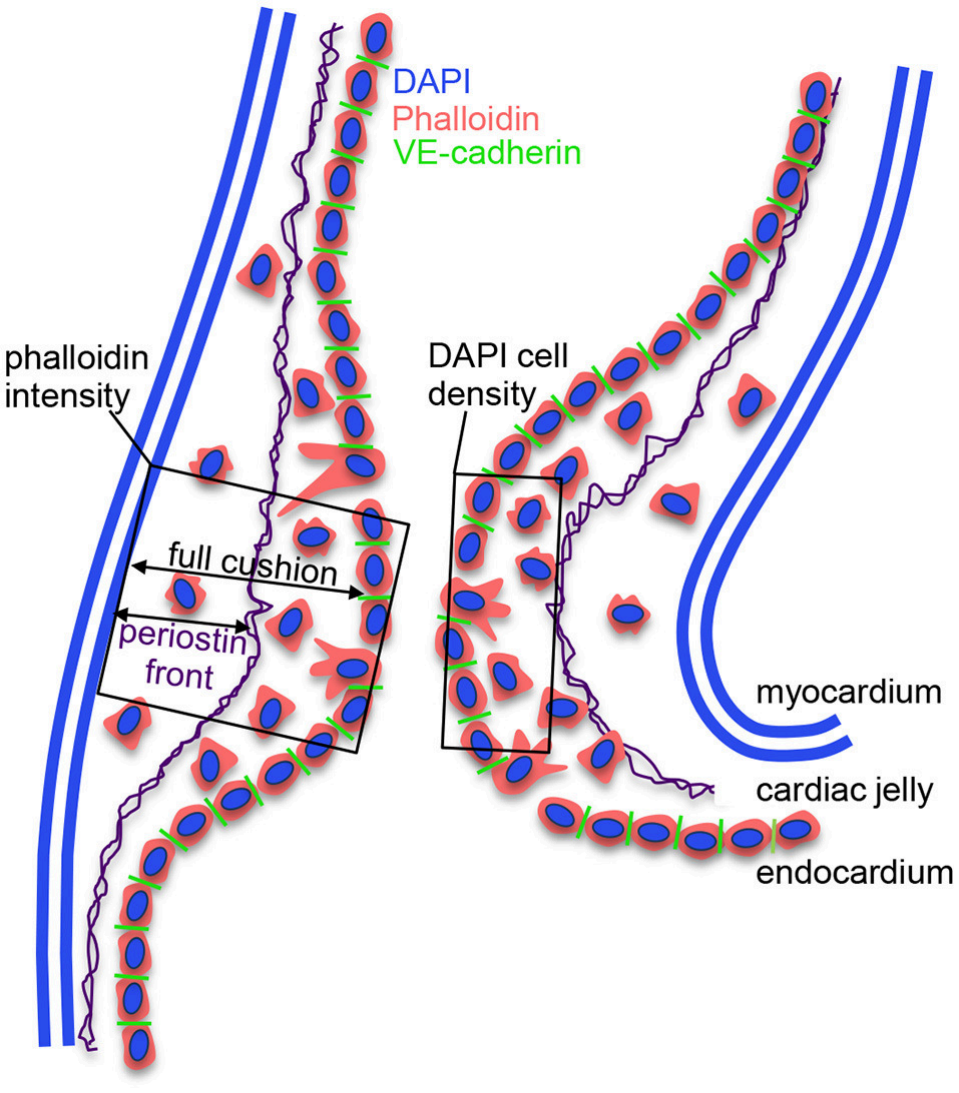
**Schematic of outflow tract cushion analysis**. The cushions are composed of an outer myocardium layer, a middle cardiac jelly layer, and an inner endocardium monolayer. VE-cadherin junctions connect endocardial cells and are lost when cells delaminate from the endocardium and invade the cardiac jelly region. Cushion cell density is measured using DAPI staining from an area that extends 10 microns into the cushion from the lumen edge, and cell organization is quantified using phalloidin staining from an area that extends across the whole width of the cushion from the lumen edge. Additionally, we measured phalloidin intensity from an area that only includes 25% of the cushion width from the lumen edge. Periostin fibers run through the cardiac jelly, where the fibers concentrate at a front that extends from the myocardium.

**Figure 2:**
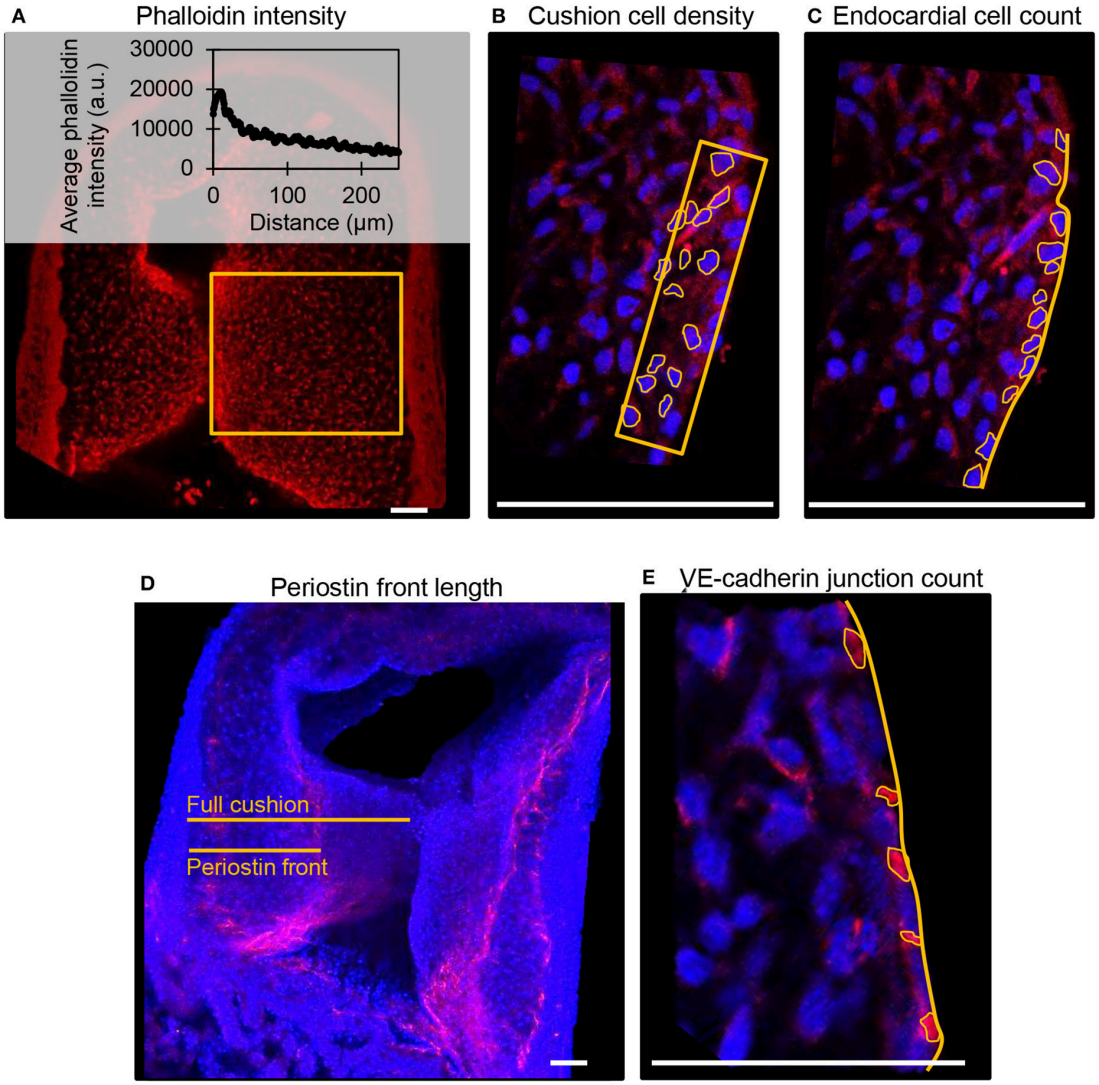
**Example confocal fluorescent image quantifications from control embryos. (A)** Phalloidin (red) stain fluorescence intensities from a full cushion, where the average fluorescent intensity at each distance position across the cushion is plotted. **(B)** Cushion cell density measured from the number of outlined DAPI-stained nuclei (blue) within an area 10 μm into the cushion from the lumen edge. **(C)** Cell count per endocardium length measured from the number of outlined DAPI-stained nuclei (blue) along with a measured length of the endocardium. **(D)** Periostin (red) front length measured as a percentage of the length of the full cushion. **(E)**. VE-cadherin (red) junction per endocardium length measured from the number of outlined junctions along a measured length of the endocardium. Scale bar = 50 microns.

Fluorescent label patterns within the outflow tract cushions were quantified from confocal images using ImageJ (NIH), and characterized based on the degree of band constriction. Figure 1 shows a schematic of the outflow tract cushion analysis. F-actin was quantified by phalloidin fluorescence intensities across the center of the outflow tract cushions, from an area that extends across the whole width of the cushion from the lumen edge and additionally from an area that only includes 25% of the cushion width from the lumen edge. DAPI fluorescence signal was used to count cells and calculate cell density from an area 10 μm into the cushions from the lumen edge and cell count per endocardium length along the endocardium edge in each embryo. Average values from three confocal slices and both cushions are reported. Cell junctions labeled with VE-cadherin antibody were counted along the endocardium edge to calculate the number of junctions per endocardium length in each embryo. Periostin localization was quantified by measuring the distance between the myocardium edge and the periostin concentration front in the cardiac jelly and calculating the percentage of the cushion width in which the periostin front extended from the myocardium. VE-cadherin and periostin quantifications were measured from the largest, centered section of the outflow tract cushions, and averaged over the inner and outer cushions. Figure 2 shows example confocal images with quantification features marked for each measure.

### Electron microscopy and quantification

Embryos with band tightness between 30 and 45% constriction were collected at HH24 (~24 h after surgical manipulation at HH18) and processed for transmission electron microscopy (TEM) and focused ion beam scanning electron microscopy (FIB-SEM). The 30–45% band tightness range corresponds to a relatively constant and large increase in peak velocity and wall shear rate (Midgett et al., 2014). TEM and FIB-SEM imaging were performed on a FEI Tecnai 12 Spirit system interfaced to an Eagle™ 2K CCD multiscan camera and a FEI Helios 660 NanoLab™ DualBeam™, respectively. TEM images were acquired as 2048 × 2048 pixel, 16-bit gray scale files using the FEI's TEM Imaging & Analysis (TIA). Whole embryos were removed from the egg and immediately immersed in fixative (2.5% paraformaldehyde + 2.5% glutaraldehyde) to maximally preserve and contract the tissue (Damon et al., 2009; Rennie et al., 2014). A portion of the outflow tract containing the cushions upstream of the banding location was then dissected from the embryo. The fixed tissue was stained and processed as previously described (Rennie et al., 2014), and oriented longitudinally in the resin block face to contain a cross-section of the outflow tract and easily locate the region of interest for imaging. One hundred nano meters sections were cut from the block face and imaged with the Tecnai™ TEM system to confirm adequate tissue contrast and fixation and identify areas of interest. The samples were then processed for Helios 660 NanoLab™ DualBeam™ FIB-SEM imaging to acquire serial (3D), high-resolution images, as previously described (Rennie et al., 2014). The FIB-SEM system alternated between imaging the block face with a scanning electron beam and milling away 10 nm thick sections with a focused ion beam, in order to collect a high-resolution 3D image volume. A maximum of 1000 slices through the endocardium were acquired, resulting in nearly the entire endocardium thickness with a region of interest width of 20 μm. Amira software was used to align the image stacks and manually segment endocardial cells in order to reconstruct and quantify the 3D cell shape and orientation.

3D endocardial cell shape and organization were quantified using a surface area-to-volume ratio, shape factor, and elongation factor for each segmented cell. Individual cells from three control and three banded samples were combined for the segmentation analysis (n_control_ = 14, n_banded_ = 26). The surface area-to-volume ratio serves as a measure of the degree of compactness and projections of each cell, and was calculated with Equation (2),

(2) $$\text{surface area}-\text{to}-\text{volume ratio}=\frac{SA}{V},$$

where *SA* is the surface area of the cell and *V* is the total volume of the segmented portion of each individual cell. The shape factor is a measure of the degree of sphericity for each cell and was calculated with Equation 3,

(3) $$\text{shape factor}=\frac{SA^{3}}{36\pi V^{2}},$$

where a perfect sphere has a shape factor of 1. Only cells with a segmented cell volume > 100 μm^3^ (n_control_ = 6, n_banded_ = 19) were included in the surface area-to-volume ratio and the shape factor analysis to avoid overly truncated segmented volumes when only small portions of cells were located within the image volume. Additionally, segmented endocardial cell volumes were quantified by an elongation factor that defines the degree of stretch of each cell using Equation 4,

(4) $$\text{elongation factor}{=}^{\lambda_{medium}}{/}_{\lambda_{largest}},$$

which is a ratio of the medium to the largest eigenvalue (λ) of the covariance matrix. A covariance matrix defines each 3D cell in space, and consists of three orthogonal eigenvectors that point in the direction that is stretched by the transformation. The eigenvalue is the factor by which the vector is stretched. Stretched objects have an elongation factor closer to zero when λ_*largest*_ is significantly larger than λ_*medium*_. Only cells that were centered in the image volume (n_control_ = 10, n_banded_ = 22) were included in the elongation factor analysis.

### Mass spectrometry

After surgical manipulations at HH18, outflow tracts were dissected from the embryo at HH24 and rinsed in ultrapure water before pooling eight outflow tracts for each sample (to get enough tissue for measurement). There were a total of five banded embryo samples (30–45% band tightness) and five sham control samples, totaling 40 outflow tracts per treatment included in these experiments. Samples were suspended in 20 μl of 50 mM ammonium bicarbonate, 0.2% ProteaseMax™ detergent (Promega, Madison, WI), shaken for 10 min, 80 μl of 50 mM ammonium bicarbonate added, and samples probe sonicated at a setting of five watts for 5 s with three repeats on ice using a Sonic Dismembrator (Fisher Scientific). The protein content of each lysate was then determined using a BCA assay (Thermo Scientific) and BSA standard, 40 μg of protein removed, and the final volume of each sample set to 80.3 μl by addition of 50 mM ammonium bicarbonate, 0.04% ProteaseMax™ solution. Samples were then reduced by addition of 1 μl of 0.5 M dithiothreitol and incubation at 56°C for 20 min, followed by alkylation by the addition of 2.7 μl of 0.55 M iodoacetamide and incubation at room temperature for 15 min. Samples were then trypsinized by the addition of 16 μl of 0.1 μg/ul sequencing grade modified trypsin (Promega) and incubation for 4 h at 37°C. Ten microliter of 10% trifluoroacetic acid was then added, samples centrifuged at 16,000 × g for 10 min, and the supernatant solid phase extracted using MicroSpin™ C18 columns (The Nest Group, Southboro, MA).

Twenty microgram portions of solid phase extracted digests were then dried by vacuum centrifugation and dissolved in 25 μl of 100 mM triethyl ammonium bicarbonate buffer. Two hundred microgram of 10-plex tandem mass tagging (TMT) reagent (Thermo Scientific) freshly dissolved in 12.5 μl of anhydrous acetonitrile (ACN) was then added to each sample and incubated at room temperature for 1 h. A short single LC-MS analysis was then performed to check for complete labeling and to determine the relative summed reporter ion intensities of each sample. This was accomplished by pooling 2 μl of each reaction mixture into 2 μl of 5% hydroxylamine, incubation at room temperature for 15 min, drying by vacuum centrifugation, dissolving in 5% formic acid, injection of 2 μg of pooled peptide mixture, and a single 2 h LC-MS run as described below. This normalization run allowed confirmation of >95% TMT labeling and generated preliminary summed reporter ion intensities for each TMT channel. Each of the original samples then had 2 μl of 5% hydroxylamine added and the 10 samples were pooled by adjusting each volume so that each would yield approximately equal summed reporter ion intensities when the final pooled mixture was analyzed by two-dimensional LC-MS as described below.

To increase numbers of quantified proteins, TMT labeled peptides were separated by two dimensions of reverse phase chromatography performed at both high and low pH. A Dionex NCS-3500RS UltiMate RSLCnano UPLC pump was used for sample loading and 2nd dimension reverse phase separation at low pH, and a Dionex NCP-3200RS UltiMate RSLCnano UPLC system for dilution of 1st dimension reverse phase eluents. Twenty microliter samples containing 50 μg of pooled TMT-labeled digest were injected for 10 min onto a NanoEase 5 μm particle XBridge BEH130 C18 300 μm × 50 mm column (Waters, Bedford, MA) at 3 μl/min in a mobile phase containing 10 mM ammonium formate (pH 9). Peptides were then eluted by sequential injection of 20 μl of 14, 17, 20, 21, 22, 23, 24, 25, 26, 27, 28, 29, 30, 35, 40, 50, and 90% ACN in 10 mM ammonium formate (pH 9) at a 3 ul/min flow rate using an autosampler. Eluted peptides were then diluted at a tee with mobile phase containing 0.1% formic acid at a 24 μl/min flow rate. Peptides were then delivered to an Acclaim PepMap 100 μm × 2 cm NanoViper C18, 5 μm particle trap on a switching valve. After 10 min of loading, the trap column was switched on-line to a PepMap RSLC C18, 2 μm, 75 μm × 25 cm EasySpray column (Thermo Scientific). Peptides were then separated at low pH in the 2nd dimension using a 7.5–30% ACN gradient in mobile phase containing 0.1% formic acid at a 300 nl/min flow rate. Each 2nd dimension LC run required 2 h for separation and reequilibration, so the entire LC/MS method required 34 h for completion. Tandem mass spectrometry data was collected using an Orbitrap Fusion Tribrid instrument configured with an EasySpray NanoSource (Thermo Scientific). Survey scans were performed in the Orbitrap mass analyzer, and data-dependent MS2 scans in the linear ion trap using collision-induced dissociation following isolation with the instrument's quadrupole. Reporter ion detection was performed in the Orbitrap mass analyzer using MS3 scans following synchronous precursor isolation of the top 10 most intense ions in the linear ion trap, and higher-energy collisional dissociation in the ion-routing multipole.

### TMT data analysis

Raw instrument files were processed using Proteome Discoverer version 1.4.1.14 (Thermo Scientific) with SEQUEST HT software and a Gallus gallus Ensembl database (release 85) containing 16,187 sequences. Searches were configured with static modifications for the TMT reagents (+229.163 peptide N-terminus and K residues) and iodoacetamide (+57.021 C residues), variable oxidation (+15.995 M residues), parent ion tolerance of +/− 1.25 Da, fragment ion tolerance of 1.0 Da, monoisotopic masses, and trypsin cleavage (max 2 missed cleavages). Searches used a reversed sequence decoy strategy to control peptide false discovery and identifications were validated using Percolator software. Only peptides with *q* ≤ 0.05, having mass errors <20 ppm, and matching only one protein entry in the database were used for reporting. Search results and TMT reporter ion intensities were exported as text files and processed using in-house scripts. After excluding the highest and lowest reporter ion intensities, an average intensity >500 was required for the remaining reporter ions to exclude the analysis of peptides with insufficient signal intensity. Reporter ions intensities with zero intensity also had values of 150 added to prevent errors during subsequent data analysis. Reporter ion intensities for peptides assigned to each protein were then summed to create an abundance measurement for each protein across the 10 samples simultaneously analyzed. Detailed instrument parameters are included in Supplemental Table 1.

### Pathway analysis

We performed a pathway analysis of our proteomics data, using Ingenuity Pathway Analysis (IPA) software (Qiagen Bioinformatics). To this end, using the acquired TMT data, we first computed protein abundances for each sample (5 control and 5 banded). These data were used to compute differential protein abundances and protein fold ratios (and their logarithms) between banded and control treatments, as well as *p*-values and false discovery rates. Protein fold ratios, *p*-values, and false discovery rates were used as input for the IPA analysis. We performed the analysis using the Ingenuity Knowledge Base, a repository of curated biological interactions and functional annotations used in IPA, by including direct and indirect relationships and endogenous chemicals, specifically for heart tissues, and with experimental observed confidence. The analysis was further performed using all node types, available data sources, and all species pathways available. IPA rendered a list of Ingenuity canonical pathways, upstream regulators, and networks that we used to further analyze our proteomics data. Further, we employed the Molecule Activity Predictor (MAP) tool available in IPA to predict the result of pathway activations/inhibitions, based on our proteomics data.

### Statistical analysis

To compare significant changes among outflow tract banded embryos compared to controls, confocal data was analyzed for the entire banded and control groups and separated based on the degree of band tightness. Linear regression analysis was used with cell density and periostin front length parameters to show band-tightness dependence. FIB-SEM samples and TMT samples were selected with a relatively tight range of band tightness (30–45%), and therefore differences between samples with varying band tightness were not analyzed. Statistical significance between banded and control groups during image analysis was determined with a two-sample Student's *t*-test, assuming significance with two-tail *p* < 0.05. Differential protein abundance was determined in the TMT experiment by comparing the summed peptide reporter ion intensities for each protein across samples (5 control, and 5 banded) using the R software (v 3.1.1) package edgeR (Robinson et al., 2010), which performed data normalization, Benjamini-Hochberg multiple test correction, and calculation of false discovery rates during tests for differential abundance for each protein compared between banded and control samples. Pathway *p*-value in IPA was calculated using the right-tailed Fisher Exact Test, and we assumed that *p* < 0.05 indicate a statistically significant, non-random association between a set of differentially regulated genes in the proteomics data and the pathway. A z-score was calculated based on the up or down-regulation of proteins in the pathway, assuming a z-score >2.0 or < −2.0 indicate significant activation or inhibition of the pathway, respectively.

## Results

### Cell infiltration occurs at different developmental times in outflow tract and atrioventricular cushions

**Figure 3:**
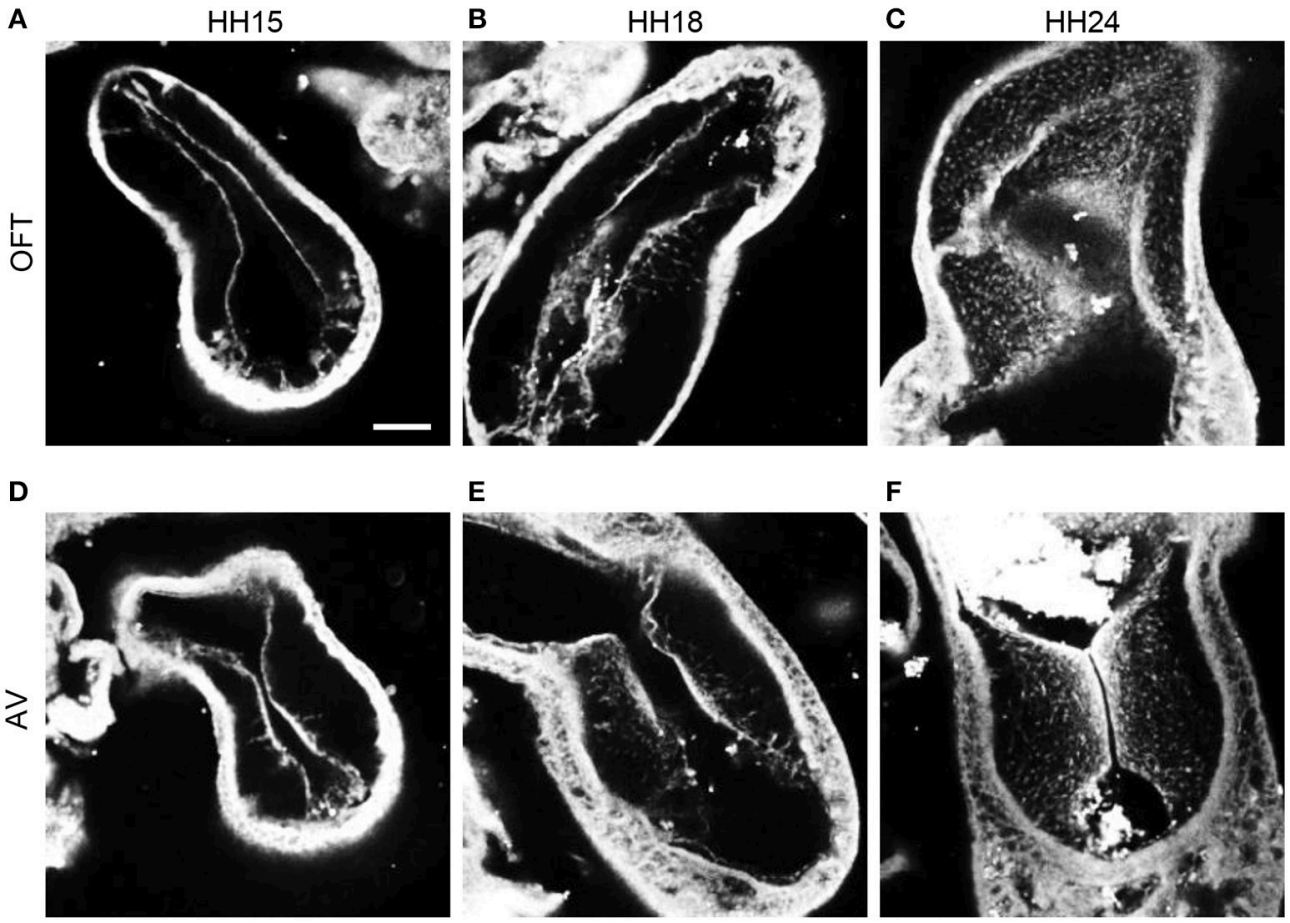
**Phalloidin confocal fluorescent image analysis**. Confocal fluorescent images at 20x from whole embryo hearts stained with phalloidin at HH15 **(A,D)**, HH18 **(B,E)**, and HH24 **(C,F)** at the outflow tract cushions **(A–C)** and the atrioventricular cushions **(D–F)**. Scale bar = 100 microns. AV, atrioventricular canal; OFT, outflow tract.

F-actin staining with phalloidin at HH15, HH18, and HH24 in both the outflow and atrioventricular segments of control hearts displayed the progression of EMT (Figure 3). At HH15, F-actin labeling extended from the endocardium into the cardiac jelly in atrioventricular cushions but not outflow tract cushions, demonstrating EMT initiation in the atrioventricular canal prior to the outflow tract. These F-actin strands are likely filopodia that extend for long distances into the cardiac jelly at the onset of EMT before invasion, as described by Kinsella and Fitzharris (1980). EMT had begun in the outflow tract by HH18 with a few strands of F-actin stain that extended from the center of the cushion endocardium, which coincided with a more disbursed configuration of F-actin staining along the entire length of the cushions in the atrioventricular canal. F-actin staining at HH24 showed an influx of cell material in outflow cushions, and a much denser layer of F-actin along the endocardium in atrioventricular cushions, indicating that EMT is more progressed in atrioventricular cushions compared to outflow tract cushions at HH24.

### Increased hemodynamic load induces changes in cardiac jelly remodeling

**Figure 4:**
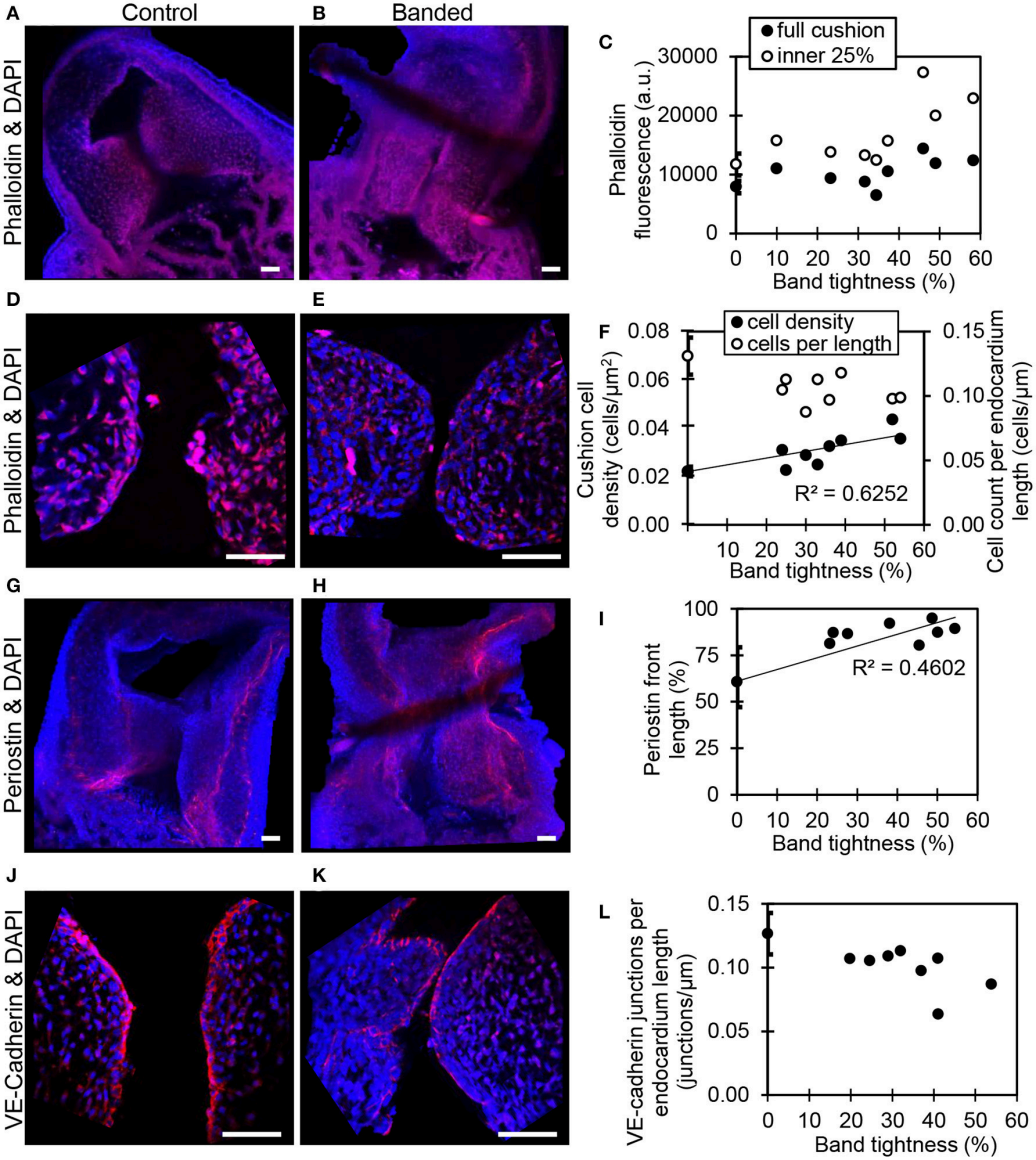
**Confocal fluorescent image analysis with banding**. Phalloidin (red) stain at 20x in a control **(A)** and banded outflow tract with 52% band tightness **(B)**, with phallodin fluorescence intensities quantitated in **(C)**. Phalloidin (red) stain at 63x in a control **(D)** and banded outflow tract with 52% band tightness **(E)**, with cushion cell density and cell count per endocardium length quantitated from DAPI stain **(F)**. Periostin (red) labeling at 20x in a control **(G)** and banded outflow tract with 49% band tightness **(H)**, with periostin front length quantitated in **(I)**. VE-cadherin (red) labeling at 63x in a control **(J)** and banded outflow tract with 41% band tightness **(K)**, with endocardial cell junctions per endocardium length quantitated in **(L)**. Each sample is also stained with DAPI (blue). 0% band tightness refers to the control group. Scale bar = 50 microns.

Confocal microscopy image analysis showed significantly more cells in outflow tract cushions of banded samples compared to controls at HH24. The largest increase in phalloidin fluorescence intensities was in the innermost 25% of the cushion adjacent to the endocardium (*p* < 0.05, *n* = 8) (Figures 4A–C). Tightly banded samples displayed a dense region of high phalloidin fluorescence intensities bordering the endocardium, which resembled the more advanced EMT phenotype displayed in atrioventricular cushions of control embryos at HH24. DAPI staining and analysis determined that cell density in the outflow tract cushion region neighboring the endocardium (an area 10 μm into the cushions from the lumen edge) was significantly increased in banded embryos compared to controls (*p* < 0.01, *n* = 8) (Figures 4D–F). Further, this cell density increased with band constriction somewhat linearly with a linear regression *R*^2^ value of 0.63, indicating that mechanisms that increase cell density after banding depend on the specific hemodynamic conditions.

**Figure 5:**
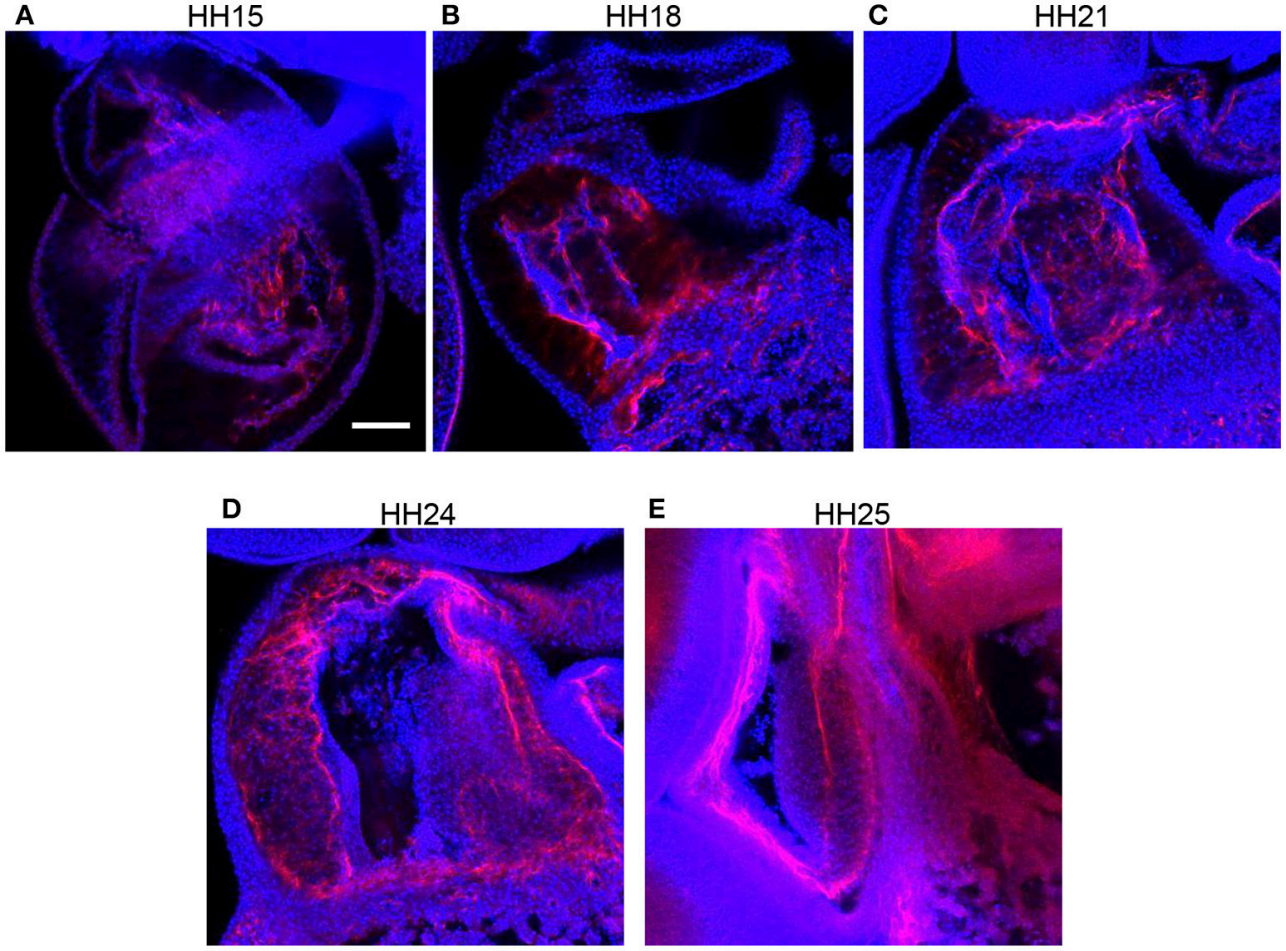
**Normal periostin expression in outflow tract cushions**. Confocal fluorescent images at 20x from whole embryo hearts labeled with periostin (red) and DAPI (blue) at progressing developmental stages, **(A)** HH15, **(B)** HH18, **(C)** HH21, **(D)** HH24, and **(E)** HH25. Scale bar = 100 microns.

**Figure 6:**
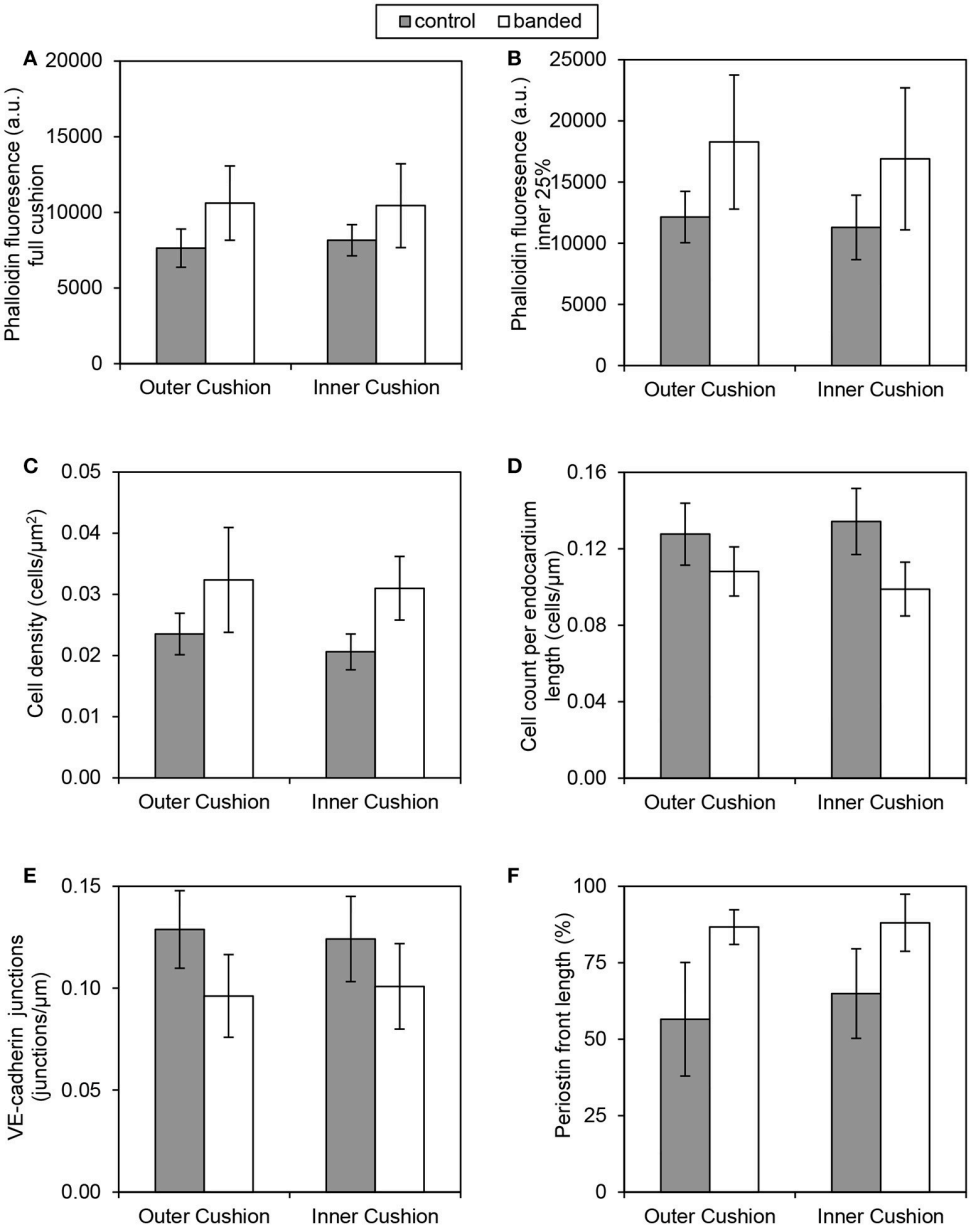
**Quantification comparisons between outer and inner cushions**. Phalloidin stain quantifications **(A,B)**, DAPI stain quantifications **(C,D)**, VE-cadherin label quantification **(E)**, and periostin label quantification **(F)**. All control and banded comparisons between outer and inner cushions were not significantly different (*p* > 0.05).

Immunohistochemistry labeling for periostin in control hearts showed a distinct fibrous periostin expression pattern which localized at the endocardium and around a subset of immediately adjacent mesenchymal cells at HH15, HH18, and HH21. In later stages at HH24 and HH25, the expression front within the outflow tract cushions ran parallel to the myocardium and endocardium layers near the middle of the outflow tract cushions (equidistant from the myocardium and endocardium) and along the cardiac jelly/myocardium interface (Figure 5). While periostin was expressed near the middle of the outflow tract cushions in control samples, the expression front instead appeared closer to the endocardium in banded samples at HH24 (Figures 4G–I). The percentage of the cushion width that the periostin front length extended from the myocardium was significantly higher in banded embryos compared to controls (*p* < 0.001, *n* = 8). The extension of the periostin front also tended to increase with band constriction, however with a low linear regression *R*^2^ value of 0.46. Since DAPI, phalloidin, and perisotin quantifications did not significantly vary between outer and inner cushions (Figure 6), all cushion data was used together to compare control and banded groups.

### Increased hemodynamic load leads to altered endocardium arrangement

Confocal microscopy analysis determined that the increased hemodynamic load after banding not only influenced cardiac jelly remodeling but also endocardium organization. There was significantly fewer DAPI stained cells along the cushion endocardium in banded embryos compared to controls at HH24 (*p* < 0.001, *n* = 8) (Figures 4D–F). Further, immunohistochemistry labeling for VE-Cadherin and confocal microscopy image analysis showed significantly fewer cell junctions along the cushion endocardium in banded embryos compared to controls (*p* < 0.01, *n* = 8) (Figures 4J–L). Tightly banded hearts had the lowest number of cell junctions per endocardium length, however, there was not a strong linear correlation between cell junctions per length and band tightness. DAPI and VE-Cadherin quantifications did not significantly vary between outer and inner cushions (Figure 6).

**Figure 7:**
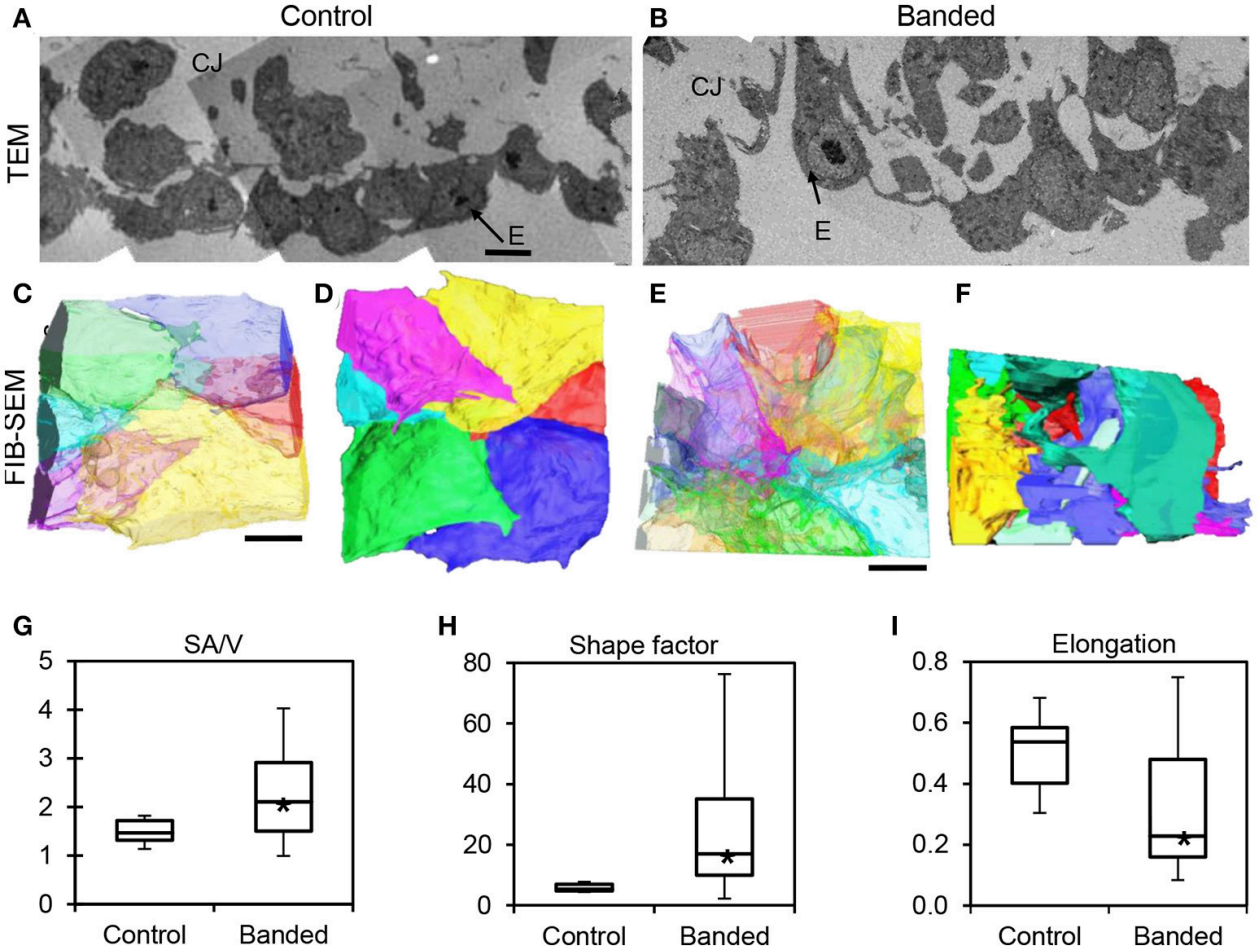
**Electron microscopy analysis of endocardium remodeling**. Stitched TEM images of the endocardium and adjacent cardiac jelly at 1700x **(A,B)**, and the corresponding 3D FIB-SEM reconstructions of endocardial cell segmentations from image stacks **(C–F)**. Reconstructions display each cell in a different color and are orientated to view the endocardial-lumen edge with translucently colored cells **(C,E)** and the endocardial-cardiac jelly interface edge with opaquely colored cells **(D,F)** from an example control embryo **(A,C,D)** and an example banded embryo **(B,E,F)**. Box plots showing differences in endocardial cell geometry, quantified from 3D segmentations of FIB-SEM images, with surface area-to-volume ratio, SA/V **(G)**, shape factor **(H)**, and elongation factor **(I)**. Boxes mark the upper and lower quartiles and whiskers mark the maximum and minimum values for each parameter. Asterisks denote statistically significant differences between experimental and control samples (*p* < 0.01). CJ, cardiac jelly; E, endocardium. Scale bar = 5 μm.

TEM images displayed a disrupted outflow tract cushion endocardium upstream of the banding site in banded embryos compared to controls at HH24. Control embryo cushions had tightly arranged endocardium with mostly plump and round cells, while banded embryo endocardial cells were more loosely organized with projections that invaded the cardiac jelly (*n* = 8) (Figures 7A,B). FIB-SEM was then used to acquire serial, high-resolution images through the endocardium and create ~0.8 mm^3^ isotropic image volumes (*n* = 3). Endocardial cell reconstructions display dramatic cell filopodia projections and chaotic layer organization in banded tissue compared to controls (Figures 7C–F). The translucent cell view of the lumen edge of the endocardium shows that control endocardial cells were tightly packed with minimal cell overlap, while the banded sample included irregular and stretched cells that overlay each other with many cell projections. The example endocardial segmentations that are oriented to view the cardiac jelly interface edge show that the control layer is mostly spherical and intact, while the banded sample interface is covered with projections that extend into the cardiac jelly. The surface area-to-volume ratio, shape factor, and elongation factor were quantified using Amira software for each 3D segmented cell volume. Box plots in Figures 7G–I show that cells from banded samples have more projections, are less spherical, and are more elongated. The average surface area-to-volume ratio and shape factor were significantly increased in banded endocardial cells compared to controls by a factor of 1.5 and 4.1, respectively (*p* < 0.01; n_control_ = 6, n_banded_ = 19). The elongation factor significantly decreased in banded endocardial cells compared to controls by a factor of 1.6 (*p* < 0.01; n_control_ = 10, n_banded_ = 22). The standard deviation of the surface area-to-volume ratio, shape factor, and elongation factor among cells was increased in the banded group compared to control by a factor of 3.1, 14.5, and 1.7, respectively.

### Increased hemodynamic load triggers EMT-related proteomic response

**Figure 8:**
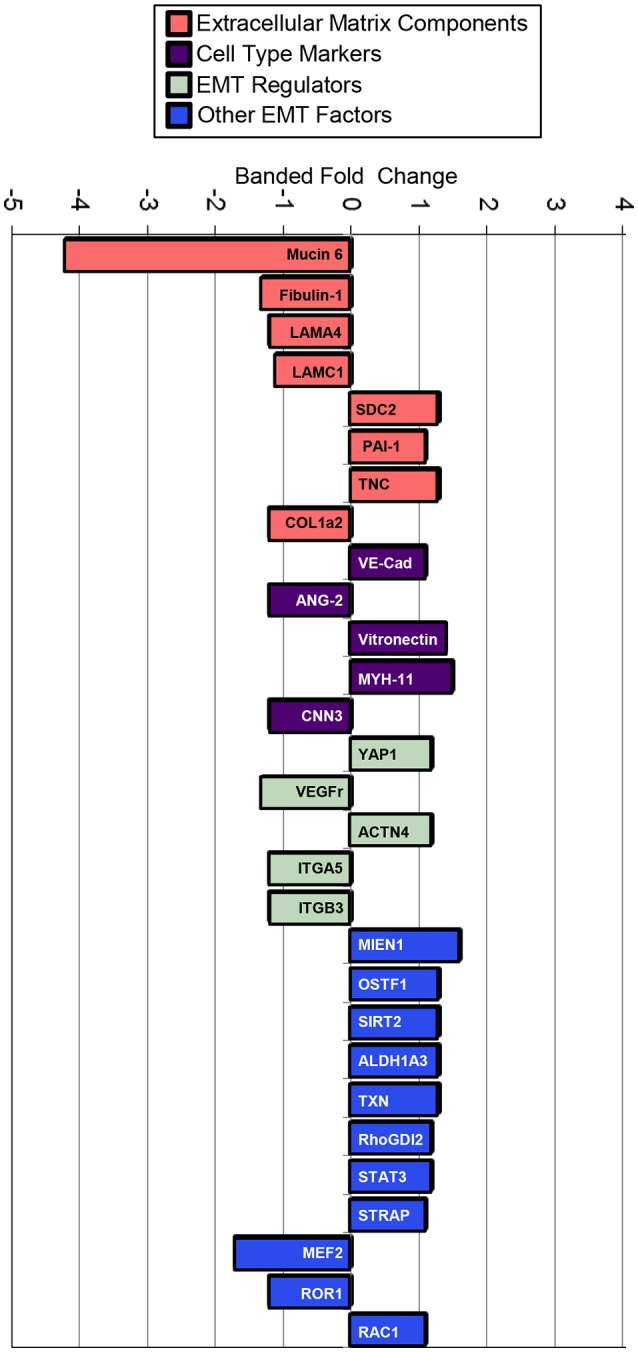
**Fold changes in mass-spectrometry measured protein abundances in banded samples compared to controls that are associated with EMT**. Extracellular matrix proteins, cell type markers, EMT regulators, and factors associated with other types of EMT are shown, with positive and negative fold changes representing protein upregulation and downregulation, respectively. Protein abbreviations are listed in Tables 1, 2.

TMT based mass spectrometry provided a proteome-wide assessment of changes in the relative abundance of specific proteins induced by increased hemodynamic load. Specifically, we examined changes in proteins known to be markers of EMT. A total of 5330 proteins were detected in the outflow tract samples, where the abundance of 606 proteins significantly differed between banded and control samples (*p* < 0.1). The full list of identified proteins, TMT reporter ion intensities, and statistical analysis are found in Supplemental Table 2, and the full proteomics data set was deposited in the PRIDE repository database (ProteomeXchange Consortium), with dataset identifier PXD005362. Of those proteins that were significantly up or downregulated, 38 proteins were related to a form of EMT, and 21 were directly related to EMT in the endocardial cushions. All EMT-related proteins had at least two peptide-spectrum matches with reporter ion signal used in quantitation among our samples, with the exception of mucin six and myocyte enhancer factor-2 that only had a single peptide for quantification. These two proteins however also had relatively high overall reporter intensity of at least 11,100 in each sample. The main protein changes include extracellular matrix components, cell type markers, and other EMT regulators (Figure 8). Table 1 lists changes in proteins related to EMT in endocardial cushions and Table 2 lists changes in proteins linked to other forms of EMT, such as in tumorigenesis and gastrulation. Each table gives the protein fold changes between control and banded samples and lists their functions related to EMT. Most fold-changes in EMT-related protein abundances are relatively small (1.2–1.5 fold), which may reflect the relatively small cushion portion of the entire outflow tract sample that is undergoing EMT.

**Table 1:** **EMT-cardiac cushion associated proteins**.

**ECM Proteins**		**Banded fold change**	***p*****-value**	**Functions**
ECM proteins	Mucin 6	−4.2x	0.01	Glycoprotein indicative of glycosaminoglycans in the heart (Coram et al., 2015), where mucins are secreted by endothelial cells and play a role in cell differentiation. Mucin 6 specifically inhibits cell adhesion to the extracellular matrix and hinders cell invasion (Leir and Harris, 2011).
	Fibulin-1	−1.3x	<0.01	Glycoprotein that suppresses EMT in the proximal outflow tract during valve morphogenesis, by repressing TGFβ2 and Snail1 levels (Harikrishnan et al., 2015).
	Laminin α 4 (LAMA4)	−1.2x	0.02	Cushion basal lamina component that stabilizes the endothelium and acts as a barrier to migratory cells (Medici and Kalluri, 2012), and becomes intermittently distributed after EMT onset (Nakajima et al., 1997).
	Laminin γ1 (LAMC1)	−1.1x	0.07	
	Syndecan 2 (SDC2)	+1.3x	0.01	Heparin sulfate proteoglycan which can concentrate glycoproteins, collagens, and growth factors ligands found in the extracellular matrix (Lockhart et al., 2011).
	Plasminogen activator 1 (PAI-1)	+1.1x	0.10	Enzyme that degrades basal lamina so that endocardial cells can invade the cardiac jelly, before other migration-inducing proteins fill the cushions (McGuire and Alexander, 1993).
	Tenascin C (TNC)	−1.3x^*^	0.01	Glycoprotein associated with cushion EMT that stimulates the TGFβ pathway (Imanaka-Yoshida and Aoki, 2014).
	Collagen 1, α2 (COL1a2)	−1.2x^*^	0.08	Type I collagen that has been associated with EMT activation (Medici and Kalluri, 2012).
Cell type markers	VE-Cadherin (VE-Cad)	+1.1x^*^	0.10	Endocardial junction marker (Person et al., 2005).
	Angiopoietin 2 (ANG-2)	−1.2x	0.04	Endothelial marker; member of the VEGF family and largely specific for vascular endothelium (Vijayaraj et al., 2012).
	Vitronectin	+1.4x	<0.01	Mesenchymal marker that is activated by Snail (Xu et al., 2009).
	Myosin-11 (MYH-11)	+1.5x	0.04	Late myogenic differentiation marker. Myosin-11 is a smooth muscle myosin belonging to the myosin heavy chain family. A portion of mesenchymal transitioned cells further differentiate into smooth muscle cells (Lin et al., 2012).
	Calponin 3 (CNN3)	−1.2x^*^	0.04	Mesenchymal marker (Moonen et al., 2015).
EMT regulators	YAP1	+1.2x	0.06	Transcription factor that promotes TGFβ-induced upregulation of Snail, Twist1, and Slug during cardiac cushion development (Zhang et al., 2014).
	Vascular endothelial growth factor receptor (VEGFr)	−1.3x	0.03	Receptor for VEGF ligand whose expression suppresses EMT during early valvulogenesis (Wang et al., 2016).
	α actinin 4 (ACTN4)	+1.2x	0.04	Microfilament protein associated with assembly and maintenance of the actin cytoskeleton in developing valves (Barnette et al., 2014), that promotes EMT through Snail signaling in cervical cancer (An et al., 2016).
	Integrin α5 (ITGA5)	−1.2x	0.03	Integrins that mediate cell-matrix adhesion, where a change in the level of expression (an integrin switch) often reflects alterations in cell-matrix interactions (Zeisberg and Neilson, 2009).
	Integrin β3 (ITGB3)	−1.2x	0.06	

* *Protein abundance change is not as expected based on increased EMT*.

**Table 2:** **Proteins associated with other types of EMT (tumorigenesis, gastrulation)**.

**Other proteins**	**Banded fold change**	***p*****-value**	**Functions**
Migration and invasion enhancer 1 (MIEN1)	+1.6x	<0.01	Promotes cell migration by inducing filopodia formation at the leading edge of migrating cells, and is predominantly overexpressed in Her-2 and luminal B subtypes of breast tumors (Kpetemey et al., 2015).
Osteoclaststimulating factor 1 (OSTF1)	+1.3x	0.01	Transcription factor that promotes migration with modulation of EMT in human embryonic kidney cells (Lai et al., 2013).
Sirtuin 2 (SIRT2)	+1.3x	<0.01	Overexpression in hepatocellular carcinoma mediates EMT by protein kinase B/glycogen synthase kinase-3β/β-catenin signaling (Chen et al., 2013).
Aldehyde dehydrogenase 1 family, member A3 (ALDH1A3)	+1.3x	0.01	Mesenchymal marker in gliomas, where ALDH1A3 overexpression was significantly associated with tumor cell invasion (Zhang et al., 2015).
Thioredoxin (TXN)	+1.3x	0.09	Mediates TGF-β-induced EMT in salivary adenoid cystic carcinoma (SACC), where higher expressions of TXN were present in human metastatic SACC compared to non-metastatic SACC tissues (Jiang et al., 2015).
Rho GDP-dissociation inhibitor 2 (RhoGDI2)	+1.2x	0.05	Overexpression stimulates EMT through the expression of Snail in gastric cancer cells, but not other family members such as Slug or Twist (Cho et al., 2014).
Signal transducer and activator Of transcription 3 (STAT3)	+1.2x	0.03	Induces TWIST1 to drive EMT and further activates AKT contributing to acquisition and metastatic maintenance (Zhao et al., 2015).
Serine/threonine kinase receptor associated protein (STRAP)	+1.1x	0.06	Promotes TGFβ-dependent EMT and metastasis of neoplastic cancer cells (Reiner and Datta, 2011).
Myocyte enhancer factor-2 (MEF2)	-1.7x^*^	0.01	Promotes EMT and invasiveness of hepatocellular carcinoma through TGFβ1 autoregulation circuitry (Yu et al., 2014).
Receptor tyrosine kinase-like orphan receptor 1 (ROR1)	-1.2x^*^	0.03	Reduces expression of E-cadherin, but enhanced the expression of SNAIL-1/2 and vimentin (Cui et al., 2013).
Ras-related C3 botulinum toxin substrate 1(RAC1)	+1.1x^*^	0.06	Small GTPase that induces EMT by regulating cell migration through control of actin polymerization (Lee et al., 2006).

* *Protein abundance change is not as expected based on increased EMT*.

### Increased hemodynamic load affects pathways related to matrix remodeling

A pathway analysis of our proteomics data using IPA software rendered a list of canonical pathways affected by banding. Out of the 5330 proteins detected, IPA mapped 3641 to known proteins/genes for pathway analysis. A total of seven canonical pathways were both significantly affected and activated/inhibited (*p* < 0.05; absolute z-score value >2.0; see Table 3). The IPA MAP tool applied to these pathways predicted inhibition of E-cadherin, and activation of cell motility, proliferation and differentiation as well as contractility (muscle cells). It also predicted, however, that the selected pathways lead to inhibition of actin nucleation and polymerization. Further, inhibition of Histone H3 was also identified, which may lead to epigenetic changes. While these pathways and predicted changes are related to EMT, no pathways directly related to EMT were listed. There were no pathways available in IPA that were directly associated with EMT in cardiac cushions during development, and the closest pathway was “Regulation of the Epithelial-Mesenchymal Transition.” The pathway was divided into four inducers of EMT: TGFβ, Notch, Wnt and Receptor Tyrosine Kinases (RTK). While the pathway was not significantly affected, after applying the MAP tool to it only the Notch-EMT and RTK-EMT pathways were predicted to render activation of EMT and inhibition of cell-cell adhesion.

**Table 3:** **Selected canonical pathways rendered by IPA**.

**Pathway**	***p*****-value**	**ratio**	**z-score**	**Function ✓ predicted response**
Remodeling of epithelial adherens junctions	1.07·10^−6^	0.70 (33/47)	−2.52	Cellular movement; cell-to-cell signaling and interaction; cellular assembly, and organization ✓ Inhibition of E-cadherin
NRF2-mediated oxidative stress response	2.07·10^−6^	0.55 (75/137)	+ 2.21	Cell death and survival; organismal survival; post-translational modification ✓ Antioxidant and detoxifying protein production
Paxillin signaling	6.25·10^−4^	0.54 (41/76)	+ 2.54	Cell morphology; cellular movement; cellular assembly and organization ✓ Activation of cell motility, proliferation, and differentiation; inhibition of cytoskeletal organization
Actin nucleation by ARP-WASP complex	7.44·10^−3^	0.55 (23/42)	−2.40^*^	Cellular assembly and organization; cellular function and maintenance; protein synthesis ✓ Inhibition of actin nucleation; activation of cell contractility
IL-22 signaling	0.013	0.65 (11/17)	+ 2.11	Cellular development; cellular growth and proliferation; hematological system development and function ✓ Activation of SOCS3 (suppressor of cytokine signaling 3)
Regulation of actin-based motility by rho	0.013	0.50 (30/60)	−2.04^*^	Cellular assembly and organization; cellular function and maintenance; tissue development ✓ Inhibition of actin nucleation and actin polymerization
UVB-induced MAPK signaling	0.022	0.50 (25/50)	+ 2.20	Cell cycle; cellular development ✓ Inhibition of histone H3 (and associated chromatin remodeling)

*Listed pathways were the most significantly affected based on p-value (< 0.05) and z-score (modulus > 2.0). The ratio reflects the fraction of molecules differentially expressed in our data vs. the total number of molecules in the pathway. The predicted response was estimated using the Molecule Activity Predictor (MAP) tool in IPA*.

* *z-score sign not as expected based on EMT increase rate*.

Since altered hemodynamic conditions trigger mechanotransduction mechanisms, we also searched for canonical networks associated with mechanotransduction processes (see Table 4). We found pathways that are usually associated with stretch and/or pressure as well as wall shear stress. While most of these pathways were significantly affected (*p* < 0.05), the highest pathway absolute z-scores were around 1.0 (rather than 2.0), indicating that, based on our data, activation/inhibition of the pathway was not significant.

**Table 4:** **Pathways associated with mechanotransduction mechanisms**.

**Pathway**	***p*****-value**	**ratio**	**z-score**	**Associated mechanotransduction**
Integrin signaling	8.07·10^−6^	0.53	−0.91	Stretch; pressure
RhoGDI signaling	2.28·10^−4^	0.52	1.16	Stretch; pressure
Signaling by rho family GTPases	5.13·10^−4^	0.48	−0.12	Stretch; pressure
Actin cytoskeleton signaling	4.93·10^−3^	0.46	0.00	Stretch; pressure
RhoA signaling	0.010	0.48	−1.12	Stretch; pressure
Tight junction signaling	0.022	0.45	−	Stretch; pressure
Renin-angiotensin signaling	0.040	0.45	−	Pressure
FAK signaling	0.068	0.44	−	Stretch; pressure
eNOS signaling	0.230	0.39	1.41	Shear stress
Gap junction signaling	0.240	0.39	−	Stretch; pressure
Endothelin-1 signaling	0.316	0.38	0.75	Shear stress

**Figure 9:**
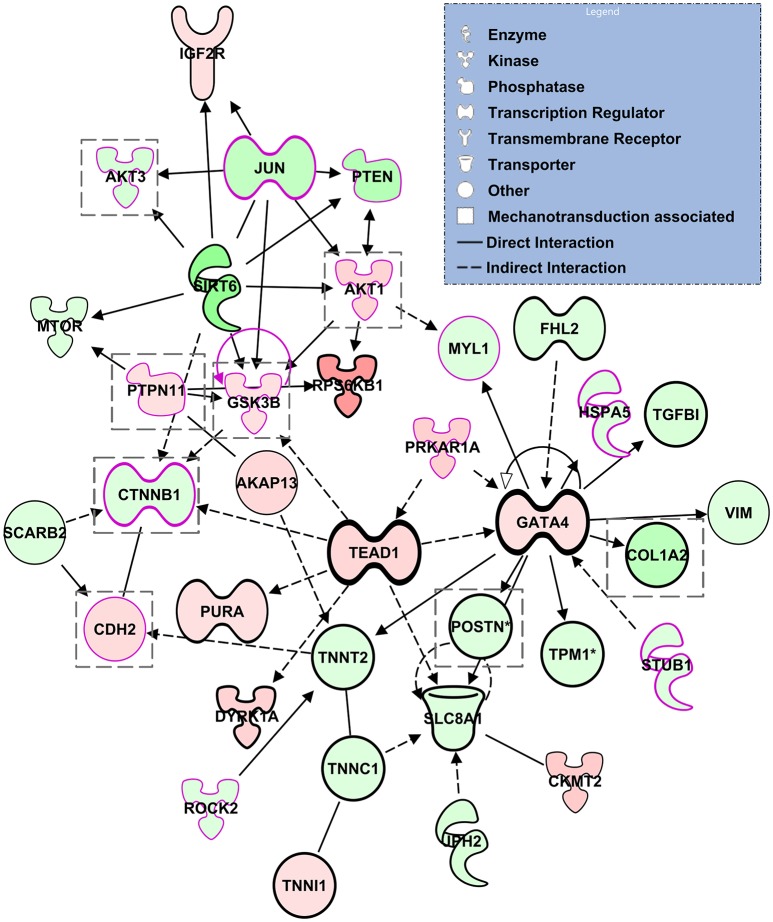
**Selected network computed by IPA**. The network show relationships between transcriptional regulators, molecules affected by mechanotransduction (highlighted with a pink borderline), and molecules related to general epithelial-mesenchymal transition (enclosed in a gray dotted square). The various shapes are associated with different types of molecules; coloring is based on differential expression values from our proteomics dataset: Red, up-regulation; Green, down-regulation. Lines indicate molecular relationships, with arrows indicating the directionality of the interaction.

We next performed a network analysis using the IPA tools. In IPA, networks are restricted to at most 35 molecules so that results can be easily visualized and further analyzed. Our proteomics data rendered 25 networks, where all networks were connected to at least one other network and thus each network represented a portion of a broader network of molecular interactions. We focused our analysis on one such network that consisted of 35 molecules all of which were differentially regulated in our data (although not necessarily significantly). Of interest, the network linked the transcriptional regulator GATA4, widely associated with CHD, with molecules related to epithelial-mesenchymal transition, and molecules that are part of mechanotransduction mechanisms (see Figure 9). Further, other genes that are also associated with CHD, such as TEAD1, CTNNB1, and PTPN11 were also part of the network. Thus the network analysis highlights the complex relationship among transcriptional regulators, EMT and mechanotransduction leading to congenital heart defects.

## Discussion

Precise spatial and temporal regulation of EMT is required for proper cardiac morphogenesis as alterations in cushion development lead to significant impairment of cardiac and valve formation and function. Previous studies have concluded that cells in mature valves are mostly derived from endothelial endocardial cushion progenitors (Combs and Yutzey, 2009). Here we used outflow tract banding to alter blood flow at the onset of EMT in the outflow tract in order to investigate the effects of increased hemodynamic load on the mesenchymal transition in the outflow tract cushions. Variations in normal EMT in the outflow tract cushions are complex to unravel *in vivo*, because of multifaceted regulation of EMT through several signaling cascades, overlapping stages of EMT activation and invasion, and the simultaneous invasion of neural crest and secondary heart field cells into the outflow tract, which are not easy to distinguish from mesenchymal cells derived from EMT. Despite these challenges, we analyzed changes in cushion cell density and EMT marker organization with confocal microscopy, characterized 3D structural variations of the endocardium assembly with FIB-SEM, and quantitated alterations in EMT-related protein abundance with mass-spectrometry after outflow tract banding. Our results taken together indicate that increased hemodynamic load enhances the EMT phenotype, which is characterized by endocardial loss of cell-cell junctions, acquisition of invasive and migratory cellular properties, and mesenchymal population of the cardiac jelly.

We employed the chicken embryo model to investigate the effects of dynamic increases in hemodynamic load produced by outflow tract banding *in vivo*, which reflect insights gained from previous *in vitro* experiments. Egorova et al. showed that increased shear stress near the outflow tract cushions activates TGFβ signaling, which is required for shear stress-induced KLF2 expression in endothelial cells (Egorova et al., 2011b). Accordingly, the elevated shear stress in our model is likely also utilizing KLF2 and TGFβ cross-talk to increase EMT. Sewell-Loftin et al. demonstrated that mechanical forces affect EMT regulation in a hydrogel explant model with varied gel deformation and strain, and determined that weaker myocardial contractions reduced atrioventricular cushion EMT (Sewell-Loftin et al., 2014). Pathway analysis reveals a possible increase in cell contractility, as well as activation of the EMT pathway through Notch, which is known to be regulated by shear stress and myocardial contractility (Masumura et al., 2009; Jahnsen et al., 2015; Samsa et al., 2015; Lee et al., 2016). Both these findings support increased EMT phenotype in outflow tract cushions. Our previous work further suggests that a compensatory pumping mechanism maintains normal stroke volume under highly elevated pressure conditions (Shi et al., 2013; Midgett et al., 2014), which may involve stronger myocardial contractions in the ventricle that may be associated with the promotion of EMT.

Confocal image analysis determined that banding induced an increase in cardiac jelly cell density and F-actin near the endocardium combined with fewer cells along the endocardial lining in the outflow tract cushions. In addition, immunohistochemistry labeling of vascular E-cadherin (VE-cadherin), an endothelial cell adherens junction protein that is lost when endocardial cells delaminate and undergo EMT (Runyan et al., 2000), showed interrupted expression along the endocardium of the outflow tract cushions in banded samples. This configuration supports an active stage of EMT when the endocardium lacks integrity of intracellular junctions and tightly connected cells, and a greater portion of endocardial cells have left the endocardium and invaded the cardiac jelly. This study also found altered expression of periostin, a TGFβ superfamily-responsive matricellular protein, that is expressed in a temporospatial pattern during cardiac development. Periostin, a marker of EMT, mediates extracellular matrix remodeling, and provides a fibrous framework for the attachment and spreading of cells undergoing EMT (Norris et al., 2004; Kern et al., 2005; Butcher et al., 2007; Lencinas et al., 2011). Periostin fibers extend through the cardiac cushions with the strongest expression concentrated at the endocardial lining of the outflow tract cushions during active stages of EMT at HH15, HH18, and HH21, and moves toward the middle of the outflow tract cushions in later developmental stages at HH24 and HH25. Our data suggests that the hemodynamics induced by outflow tract banding alter periostin expression patterns so that the fibers are organized closer to the endocardium in banded samples compared to controls at HH24. This change indicates that increased hemodynamic load induces an active stage of EMT in the outflow tract at HH24. Transient expression of periostin and other EMT markers like fibroblast-specific protein 1 and Snail (Zeisberg and Neilson, 2009), deserves further investigation to determine precise dynamic changes in EMT during altered hemodynamics between HH18 and HH24.

3D FIB-SEM endocardial cell segmentations of banded samples correspond with previous electron microscopy characterizations of the morphologic changes of cells undergoing EMT, while controls display a more immature endocardium configuration (Markwald et al., 1975; Clark et al., 1989; Icardo, 1989). Structural descriptions of EMT detail how a portion of endocardial cells in the cardiac cushions lose their orientation as individual cells change shape, flatten, and extend filopodial and lamellipodial projections over neighboring endocardial cells and into the cardiac jelly. As cells migrate into the cardiac jelly, the remaining endocardial cells reorganize and establish new contacts to restore an intact layer. These hallmarks of EMT are displayed in TEM and FIB-SEM images of our banded samples, where overall quantified changes in the average cell surface area-to-volume ratio, shape factor, and elongation from FIB-SEM data sets suggest that banding increases EMT in the outflow tract cushions. Large standard deviations associated with the quantification measures of cells from banded embryos compared to controls reflect that only a portion of endocardial cells undergo EMT and thus vary in shape and organization. Even though the entire volume of each cell was rarely acquired within the FIB-SEM image volume, the small standard deviations of the control cells compared to the banded show that the quantification measures detected valid differences in cell shape and elucidate the ways in which the endocardial cells change after banding.

We used mass spectrometry to detect global changes in protein abundances after banding to reflect the overall dynamic regulation balance between transcription, localization, modification and programmed destruction of protein during EMT. The proteomic analysis corroborates the confocal and FIB-SEM evaluations in this manuscript, further indicating that the hemodynamics induced by banding enhances EMT in the outflow tract. The TMT results do not show significant changes in TGFβ ligand between control and banded samples. However, the EMT-related proteins that are up or downregulated in banded samples include extracellular matrix components, cell type markers, and EMT transcription factors, indicating that perhaps the increased hemodynamic load interferes with EMT signaling pathways downstream of growth factor binding. The compositional extracellular matrix reorganization in banded samples promotes EMT cell migration and TGFβ regulation, with an overall decrease in early matrix components that inhibit EMT and stabilize the endocardium (mucin, laminin, and fibulin). This remodeling may encourage cell migration along EMT-stimulating matrix components, such as hyaluronic acid and versican, and allow for more efficient secretion and binding of growth factors released from the myocardium. Overall, significant changes in cell type marker protein counts in banded samples compared to controls also correlate with increased EMT, with decreased endothelial markers (angiopoietin 2) and increased mesenchymal markers (vitronectin, myosin-11). Small changes and discrepancies in endothelial markers, such as a slight increase in VE-Cadherin protein in banded samples (+1.1x, *p* = 0.1) may reflect the relatively small portion of endocardial cells in the whole outflow tract samples. The small increase in VE-cadherin protein abundance may also reflect the reformation of cell-cell adherens junctions between the majority of cells that remain in the endocardial layer after EMT. The other EMT regulatory proteins that are altered in banded samples (YAP1, VEGFr, α actinin 4, integrins) promote EMT through TGFβ and VEGF pathways. These changes in EMT-related proteins deserve further study to determine how each component may contribute to the increased EMT phenotype in the outflow tract cushions after banding. For example, while YAP1 showed a trend toward elevated expression in the banded samples, a characterization of its phosphorylation state would establish whether the increased expression is correlated with its control of cell proliferation and therefore increased cell counts in the outflow tract cushions (Lamar et al., 2012; Shao et al., 2014). Even though the protein variations listed in Table 2 have only been implicated in other types of EMT, such as in tumorigenesis and gastrulation, these changes are likely associated with cardiac cushion EMT in the banded samples since major elements of EMT regulation are reiterated among various embryonic development events and adult pathologies. The majority of these protein changes linked with other types of EMT promote EMT through TGFβ and Wnt/β-catenin pathways. Endocardial cushion formation can be induced by TGFβ and Wnt/β-catenin signaling, with Notch signaling also playing an important role in endocardial cushion EMT (Combs and Yutzey, 2009). While there were no significant changes in TGFβ, Wnt, or Notch in the TMT analysis, observed changes indicate that increased hemodynamic load alters the downstream EMT proteins regulated by these signaling pathways.

Pathway analysis further revealed intricate relationships among EMT, cardiac transcription factors, and mechanotransduction in response to increased hemodynamic load. In fact, our analysis confirmed inhibition of cell-cell adhesion, required for EMT initiation in the endothelium, as well as extracellular matrix remodeling including cell motility and proliferation required to populate the cushions. Further, many pathways associated with hemodynamic load mechanotransduction were also significantly differentially regulated.

Interestingly network analysis of our proteomics data suggests links among hemodynamic load, EMT signaling, and recognized key players in CHD including PTPN11, as well as cardiac transcriptional regulators, such as GATA4, TEAD1, and CTNNB1. GATA4 is a cardiac transcription factor required for cardiac development. GATA4 interacts with other transcription factors, including NKX2.5, TBX5, and MEF2A to regulate heart development and gene expression (Nimura and Kaneda, 2016). While GATA4 knockdown is embryolethal, mutations of GATA4 are associated with cardiac septal defects as well as pulmonary stenosis (Garg et al., 2003; Tomita-Mitchell et al., 2007; McCulley and Black, 2012; Misra et al., 2012), but also with tetralogy of Fallot (TOF) and double outlet right ventricle (DORV) (Andersen et al., 2014). TEAD1 (TEA domain family member 1, also known as transcriptional enhancer factor 1, TEF-1), a cardiac transcription factor that regulates muscle gene expression, interacts with GATA4 and other transcription factors, including NKX2.5 (He et al., 2011; Nimura and Kaneda, 2016). In addition, CTNNB1 (a downstream component of the Wnt signaling pathway) encodes a protein that forms adherens junctions, and thus is necessary for the creation and maintenance of epithelial and endothelial cell layers. Inhibition of CTNNB1, therefore, leads to inhibition of adherens junctions in the outflow tract endocardial cushions. *De novo* mutations of CTNNB1 have been found in CHD cases that also present neurodevelopmental disorders (Homsy et al., 2015). Finally, PTPN11 has also been implicated in CHD (Homsy et al., 2015). The PTPN11 genes encodes the protein SHP2, a regulatory component of the RAS/MAPK signaling pathway, which is involved in cell differentiation, migration and apoptosis (Combs and Yutzey, 2009). PTPN11 mutations cause approximately 50% of Noonan syndrome, an autosomal dominant disorder associated with several congenital cardiac defects, including pulmonary stenosis, hypertrophic cardiomyopathy, and atrioventricular septal defects (Sarkozy et al., 2003; Weismann et al., 2005). PTPN11 gene mutations have also been found in multiple lentigines (leopard) syndrome, also associated with pulmonary stenosis (Sarkozy et al., 2003). Mice with activating SHP2 mutations in the endocardial cell lineage have increased proliferation of OFT endocardial cushion endothelial and mesenchymal cells, leading to cardiac malformations such as ventricular septal defects and DORV (Krenz et al., 2008; Combs and Yutzey, 2009). Overall these findings recapitulate and explain cardiac defects observed after banding: mainly ventricular septal defects, TOF, and DORV (Midgett and Rugonyi, 2014; Midgett et al., 2017). These relationships could help explain why embryos with altered blood flow develop cardiac defects similar to those seen in babies with genetic anomalies, and why many babies with congenital heart defects do not seem to present genetic anomalies.

Recently, Menon et al created 3D hematoxylin and eosin image reconstructions of the outflow tract after banding the ventricle/outflow tract junction at HH16-17, and measured decreased cushion volume with fewer cells in the cardiac jelly after banding (*n* = 3) (Menon et al., 2015). There are a few key differences between this report and the current study that may explain the varied cushion remodeling outcomes. First, Menon et al banded the outflow tract *ex ovo* at HH16-17 instead of at HH18. Second and likely most importantly, their results reflect the effects of altered hemodynamics downstream of the outflow tract/ventricle junction band site, while our study investigated cushion remodeling upstream of the mid-section outflow tract band site. This is due to different placement of the band in each study. The hemodynamic variations due to band placement may account for the differences in the number of cells in the outflow tract cushions.

Taken together, our imaging and proteomic data indicate that the hemodynamic forces induced by banding interfere with normal developmental cushion formation by increasing EMT in the outflow tract cushions. While our analysis did not discriminate between mesenchymal cells arising from EMT and those derived from the neural crest or proliferation, increased cell density in the cardiac jelly was accompanied by a loosely packed endocardium in phalloidin and DAPI stained confocal images, VE-cadherin junction disruption in immunohistochemically labeled confocal images, elongated and less spherical endocardial cells with more projections in FIB-SEM image volumes, and periostin expression closer to the endocardium in immunohistochemically labeled confocal images. In addition, our proteomic analysis indicated a reorganization of the extracellular matrix and increased TGFβ-related signaling. Proteomics pathway analysis confirmed extracellular matrix reorganization and EMT phenotype and pointed to interesting and intricate relationships among mechanotransduction pathways and EMT. While outside the scope of this study, further investigations are underway to characterize the time course of transient changes in gene expression that result in increased EMT at HH24 after outflow tract banding at HH18. Heart development is a finely regulated process with several remodeling mechanisms acting in parallel during the looping heart stages. This study indicates that EMT is one of the initial processes affected by increased hemodynamic load that triggers a detrimental cascade of events that eventually leads to cardiac defects.

## Author contributions

MM coordinated the study, carried out a large portion of the data collection and data analysis, and drafted the manuscript; CL carried out the work done in the Multiscale Microscopy Core in the OHSU Center for Spatial Systems Biomedicine and contributed to the manuscript; LD carried out the work and data analysis done in the OHSU Proteomics Core and contributed to the manuscript; AM supervised the pathway analysis and edited the manuscript; SR conceived of the study and helped draft the manuscript.

## Funding

This work was funded in part by a grant from US National Institutes of Health, NIH R01HL094570, and NIH F31 HL129684, as well as a grant from the American Heart Association grant, 16PRE31180006. The electron microscopy studies were partially funded by an internal pilot project award, OHSU Center for Spatial Systems Biomedicine award. The proteomics work was partially supported by an internal pilot grant, OHSU School of Medicine Faculty Innovation Fund, and NIH grants P30EY01572, P30CA069533, and S10OD012246.

### Conflict of interest statement

The authors declare that the research was conducted in the absence of any commercial or financial relationships that could be construed as a potential conflict of interest.

## Abbreviations

CHD: congenital heart disease
DORV: double outlet right ventricle
EMT: endothelial–mesenchymal transition
FIB-SEM: focused ion beam scanning electron microscopy
HH: Hamburger and Hamilton development stage
IPA: Ingenuity Pathway Analysis
KLF2: Krüppel like factor 2
MAP: Molecule Activity Predictor (IPA tool)
OCT: optical coherence tomography
TEM: transmission electron microscopy
TGFβ: transforming growth factor beta
TMT: tandem mass tagging
TOF: tetralogy of Fallot
VEGF: vascular endothelial growth factor
Erbb3, Receptor tyrosine-protein kinase erbB-3: also known as HER3 (human epidermal growth factor receptor 3).

## References

[B1] An H.-T. Yoo S. Ko J. (2016). α-Actinin-4 induces the epithelial-to-mesenchymal transition and tumorigenesis via regulation of Snail expression and β-catenin stabilization in cervical cancer. Oncogene 35, 5893–5904. 10.1038/onc.2016.117 27065319

[B2] Andersen T. A. de Troelsen K. L. Larsen L. A. (2014). Of mice and men: molecular genetics of congenital heart disease. Cell. Mol. Life Sci. 71, 1327–1352. 10.1007/s00018-013-1430-1 23934094 PMC3958813

[B3] Armstrong E. J. Bischoff J. (2004). Heart valve development: endothelial cell signaling and differentiation. Circ. Res. 95, 459–470. 10.1161/01.RES.0000141146.95728.da 15345668 PMC2810618

[B4] Barnette D. N. VandeKopple M. Wu Y. Willoughby D. A. Lincoln J. (2014). RNA-seq analysis to identify novel roles of scleraxis during embryonic mouse heart valve remodeling. PLoS ONE 9:e101425. 10.1371/journal.pone.0101425 24983472 PMC4077804

[B5] Butcher J. T. Norris R. A. Hoffman S. Mjaatvedt C. H. Markwald R. R. (2007). Periostin promotes atrioventricular mesenchyme matrix invasion and remodeling mediated by integrin signaling through Rho/PI 3-kinase. Dev. Biol. 302, 256–266. 10.1016/j.ydbio.2006.09.048 17070513 PMC1913192

[B6] Chen J. Chan A. W. To K. F. Chen W. Zhang Z. Ren J.. (2013). SIRT2 overexpression in hepatocellular carcinoma mediates epithelial to mesenchymal transition by protein kinase B/glycogen synthase kinase-3β/β-catenin signaling. Hepatology 57, 2287–2298. 10.1002/hep.26278 23348706

[B7] Cho H. J. Park S. M. Kim I. K. Nam I. K. Baek K. E. Im M. J.. (2014). RhoGDI2 promotes epithelial-mesenchymal transition via induction of Snail in gastric cancer cells. Oncotarget 5, 1554–1564. 10.18632/oncotarget.1733 24721928 PMC4039231

[B8] Clark E. B. Hu N. Frommelt P. Vandekieft G. K. Dummett J. L. Tomanek R. J. (1989). Effect of increased pressure on ventricular growth in stage 21 chick embryos. Am. J. Physiol. 257(1 Pt 2), H55–H61. 2750949 10.1152/ajpheart.1989.257.1.H55

[B9] Clark E. B. Rosenquist G. C. (1978). Spectrum of cardiovascular anomalies following cardiac loop constriction in the chick embryo. Birth Defects Orig. Artic. Ser. 14, 431–442. 737312

[B10] Combs M. D. Yutzey K. E. (2009). Heart valve development: regulatory networks in development and disease. Circ. Res. 105, 408–421. 10.1161/CIRCRESAHA.109.201566 19713546 PMC2777683

[B11] Coram R. J. Stillwagon S. J. Guggilam A. Jenkins M. W. Swanson M. S. Ladd A. N. (2015). Muscleblind-like 1 is required for normal heart valve development *in vivo*. BMC Dev. Biol. 15:36. 10.1186/s12861-015-0087-4 26472242 PMC4608261

[B12] Cui B. Zhang S. Chen L. Yu J. Widhopf G. F. II Fecteau J. F.. (2013). Targeting ROR1 inhibits epithelial-mesenchymal transition and metastasis. Cancer Res. 73, 3649–3660. 10.1158/0008-5472.can-12-3832 23771907 PMC3832210

[B13] Culver J. C. Dickinson M. E. (2010). The effects of hemodynamic force on embryonic development. Microcirculation 17, 164–178. 10.1111/j.1549-8719.2010.00025.x 20374481 PMC2927969

[B14] Damon B. J. Remond M. C. Bigelow M. R. Trusk T. C. Xie W. Perucchio R.. (2009). Patterns of muscular strain in the embryonic heart wall. Dev. Dyn. 238, 1535–1546. 10.1002/dvdy.21958 19418446 PMC2757264

[B15] Davies P. F. (1995). Flow-mediated endothelial mechanotransduction. Physiol. Rev. 75, 519–560. 7624393 10.1152/physrev.1995.75.3.519 PMC3053532

[B16] Egorova A. D. Khedoe P. P. Goumans M. J. Yoder B. K. Nauli S. M. ten Dijke P.. (2011a). Lack of primary cilia primes shear-induced Endothelial-to-Mesenchymal Transition. Circ. Res. 108, 1093–1101. 10.1161/CIRCRESAHA.110.231860 21393577 PMC3094764

[B17] Egorova A. D. Van der Heiden K. Van de Pas S. Vennemann P. Poelma C. DeRuiter M. C.. (2011b). Tgfbeta/Alk5 signaling is required for shear stress induced klf2 expression in embryonic endothelial cells. Dev. Dyn. 240, 1670–1680. 10.1002/dvdy.22660 21604321

[B18] Eisenberg L. M. Markwald R. R. (1995). Molecular regulation of atrioventricular valvuloseptal morphogenesis. Circ. Res. 77, 1–6. 7788867 10.1161/01.res.77.1.1

[B19] Garg V. Kathiriya I. S. Barnes R. Schluterman M. K. King I. N. Butler C. A.. (2003). GATA4 mutations cause human congenital heart defects and reveal an interaction with TBX5. Nature 424, 443–447. 10.1038/nature01827 12845333

[B20] Gitler A. D. Lu M. M. Jiang Y. Q. Epstein J. A. Gruber P. J. (2003). Molecular markers of cardiac endocardial cushion development. Dev. Dyn. 228, 643–650. 10.1002/dvdy.10418 14648841

[B21] Gittenberger-de Groot A. C. Bartelings M. M. Deruiter M. C. Poelmann R. E. (2005). Basics of cardiac development for the understanding of congenital heart malformations. Pediatr. Res. 57, 169–176. 10.1203/01.pdr.0000148710.69159.61 15611355

[B22] Hamburger V. Hamilton H. L. (1992). A series of normal stages in the development of the chick embryo. 1951. Dev. Dyn. 195, 231–272. 10.1002/aja.1001950404 1304821

[B23] Harikrishnan K. Cooley M. A. Sugi Y. Barth J. L. Rasmussen L. M. Kern C. B.. (2015). Fibulin-1 suppresses endothelial to mesenchymal transition in the proximal outflow tract. Mech. Dev. 136, 123–132. 10.1016/j.mod.2014.12.005 25575930 PMC4868094

[B24] He A. Kong S. W. Ma Q. Pu W. T. (2011). Co-occupancy by multiple cardiac transcription factors identifies transcriptional enhancers active in heart. Proc. Natl. Acad. Sci. U.S.A. 108, 5632–5637. 10.1073/pnas.1016959108 21415370 PMC3078411

[B25] Hinton R. B. Jr. Lincoln J. Deutsch G. H. Osinska H. Manning P. B. Benson D. W.. (2006). Extracellular matrix remodeling and organization in developing and diseased aortic valves. Circ. Res. 98, 1431–1438. 10.1161/01.RES.0000224114.65109.4e 16645142

[B26] Hinton R. B. Yutzey K. E. (2011). Heart valve structure and function in development and disease. Annu. Rev. Physiol. 73, 29–46. 10.1146/annurev-physiol-012110-142145 20809794 PMC4209403

[B27] Hoffman J. I. Kaplan S. (2002). The incidence of congenital heart disease. J. Am. Coll. Cardiol. 39, 1890–1900. 10.1016/S0735-1097(02)01886-7 12084585

[B28] Hogers B. DeRuiter M. C. Gittenberger-de Groot A. C. Poelmann R. E. (1997). Unilateral vitelline vein ligation alters intracardiac blood flow patterns and morphogenesis in the chick embryo. Circ. Res. 80, 473–481. 9118477 10.1161/01.res.80.4.473

[B29] Hogers B. DeRuiter M. C. Gittenberger-de Groot A. C. Poelmann R. E. (1999). Extraembryonic venous obstructions lead to cardiovascular malformations and can be embryolethal. Cardiovasc. Res. 41, 87–99. 10325956 10.1016/s0008-6363(98)00218-1

[B30] Homsy J. Zaidi S. Shen Y. Ware J. S. Samocha K. E. Karczewski K. J.. (2015). *De novo* mutations in congenital heart disease with neurodevelopmental and other birth defects. Science 350, 1262–1266. 10.1126/science.aac9396 26785492 PMC4890146

[B31] Hove J. R. Koster R. W. Forouhar A. S. Acevedo-Bolton G. Fraser S. E. Gharib M. (2003). Intracardiac fluid forces are an essential epigenetic factor for embryonic cardiogenesis. Nature 421, 172–177. 10.1038/nature01282 12520305

[B32] Hu N. Christensen D. A. Agrawal A. K. Beaumont C. Clark E. B. Hawkins J. A. (2009). Dependence of aortic arch morphogenesis on intracardiac blood flow in the left atrial ligated chick embryo. Anat. Rec. (Hoboken) 292, 652–660. 10.1002/ar.20885 19322826

[B33] Icardo J. (1989). Changes in endocardial cell morphology during development of the endocardial cushions. Anat. Embryol. 179, 443–448. 10.1007/BF00319586 2729607

[B34] Imanaka-Yoshida K. Aoki H. (2014). Tenascin-C and mechanotransduction in the development and diseases of cardiovascular system. Front. Physiol. 5:283. 10.3389/fphys.2014.00283 25120494 PMC4114189

[B35] Jahnsen E. D. Trindade A. Zaun H. C. Lehoux S. Duarte A. Jones E. A. V. (2015). Notch1 is pan-endothelial at the onset of flow and regulated by flow. PLoS ONE 10:e0122622. 10.1371/journal.pone.0122622 25830332 PMC4382190

[B36] Jiang Y. Feng X. Zheng L. Li S.-L. Ge X.-Y. Zhang J.-G. (2015). Thioredoxin 1 mediates TGF-β-induced epithelial-mesenchymal transition in salivary adenoid cystic carcinoma. Oncotarget 6, 25506–25519. 10.18632/oncotarget.4635 26325518 PMC4694848

[B37] Kern C. B. Hoffman S. Moreno R. Damon B. J. Norris R. A. Krug E. L.. (2005). Immunolocalization of chick periostin protein in the developing heart. Anat. Rec. A Discov. Mol. Cell. Evol. Biol. 284, 415–423. 10.1002/ar.a.20193 15803479

[B38] Kinsella M. G. Fitzharris T. P. (1980). Origin of cushion tissue in the developing chick heart: cinematographic recordings of *in situ* formation. Science 207, 1359–1360. 7355294 10.1126/science.7355294

[B39] Kpetemey M. Dasgupta S. Rajendiran S. Das S. Gibbs L. D. Shetty P.. (2015). MIEN1, a novel interactor of Annexin A2, promotes tumor cell migration by enhancing AnxA2 cell surface expression. Mol. Cancer 14, 156. 10.1186/s12943-015-0428-8 26272794 PMC4536591

[B40] Krenz M. Gulick J. Osinska H. E. Colbert M. C. Molkentin J. D. Robbins J. (2008). Role of ERK1/2 signaling in congenital valve malformations in Noonan syndrome. Proc. Natl. Acad. Sci. U.S.A. 105, 18930–18935. 10.1073/pnas.0806556105 19017799 PMC2596231

[B41] Lai K. P. Law A. Y. Lau M. C. Takei Y. Tse W. K. Wong C. K. (2013). Osmotic stress transcription factor 1b (Ostf1b) promotes migration properties with the modulation of epithelial mesenchymal transition (EMT) phenotype in human embryonic kidney cell. Int. J. Biochem. Cell Biol. 45, 1921–1926. 10.1016/j.biocel.2013.05.023 23732111

[B42] Lamar J. M. Stern P. Liu H. Schindler J. W. Jiang Z.-G. Hynes R. O. (2012). The Hippo pathway target, YAP, promotes metastasis through its TEAD-interaction domain. Proc. Natl. Acad. Sci. U.S.A. 109, E2441–E2450. 10.1073/pnas.1212021109 22891335 PMC3443162

[B43] Lee J. Fei P. Packard R. R. S. Kang H. Xu H. Baek K. I.. (2016). 4-Dimensional light-sheet microscopy to elucidate shear stress modulation of cardiac trabeculation. J. Clin. Invest. 126, 1679–1690. 10.1172/JCI83496 27018592 PMC4855946

[B44] Lee J. M. Dedhar S. Kalluri R. Thompson E. W. (2006). The epithelial-mesenchymal transition: new insights in signaling, development, and disease. J. Cell Biol. 172, 973–981. 10.1083/jcb.200601018 16567498 PMC2063755

[B45] Leir S. H. Harris A. (2011). MUC6 mucin expression inhibits tumor cell invasion. Exp. Cell Res. 317, 2408–2419. 10.1016/j.yexcr.2011.07.021 21851820

[B46] Lencinas A. Tavares A. L. Barnett J. V. Runyan R. B. (2011). Collagen gel analysis of epithelial-mesenchymal transition in the embryo heart: an *in vitro* model system for the analysis of tissue interaction, signal transduction, and environmental effects. Birth Defects Res. C Embryo Today 93, 298–311. 10.1002/bdrc.20222 22271679

[B47] Lim J. Thiery J. P. (2012). Epithelial-mesenchymal transitions: insights from development. Development 139, 3471–3486. 10.1242/dev.071209 22949611

[B48] Lin F. Wang N. Zhang T. C. (2012). The role of endothelial-mesenchymal transition in development and pathological process. IUBMB Life 64, 717–723. 10.1002/iub.1059 22730243

[B49] Liu A. Yin X. Shi L. Li P. Thornburg K. L. Wang R.. (2012). Biomechanics of the Chick embryonic heart outflow tract at HH18 using 4D optical coherence tomography imaging and computational modeling. PLoS ONE 7:e40869. 10.1371/journal.pone.0040869 22844414 PMC3402486

[B50] Lockhart M. Wirrig E. Phelps A. Wessels A. (2011). Extracellular matrix and heart development. Birth Defects Res. A Clin. Molteratol. 91, 535–550. 10.1002/bdra.20810 21618406 PMC3144859

[B51] Ma Z. Liu A. Yin X. Troyer A. Thornburg K. Wang R. K.. (2010). Measurement of absolute blood flow velocity in outflow tract of HH18 chicken embryo based on 4D reconstruction using spectral domain optical coherence tomography. Biomed. Opt. Express 1, 798–811. 10.1364/BOE.1.000798 21127734 PMC2994554

[B52] Markwald R. R. Fitzharris T. P. Smith W. N. (1975). Sturctural analysis of endocardial cytodifferentiation. Dev. Biol. 42, 160–180. 1112439 10.1016/0012-1606(75)90321-8

[B53] Masumura T. Yamamoto K. Shimizu N. Obi S. Ando J. (2009). Shear Stress increases expression of the arterial endothelial marker EphrinB2 in murine ES cells via the VEGF-Notch signaling pathways. Arterioscler. Thromb. Vasc. Biol. 29, 2125–2131. 10.1161/atvbaha.109.193185 19797707

[B54] McCulley D. J. Black B. L. (2012). Transcription factor pathways and congenital heart disease. Curr. Top. Dev. Biol. 100, 253–277. 10.1016/B978-0-12-387786-4.00008-7 22449847 PMC3684448

[B55] McGuire P. G. Alexander S. M. (1993). Inhibition of urokinase synthesis and cell surface binding alters the motile behavior of embryonic endocardial-derived mesenchymal cells *in vitro*. Development 118, 931–939. 8076527 10.1242/dev.118.3.931

[B56] McQuinn T. C. Bratoeva M. Dealmeida A. Remond M. Thompson R. P. Sedmera D. (2007). High-frequency ultrasonographic imaging of avian cardiovascular development. Dev. Dyn. 236, 3503–3513. 10.1002/dvdy.21357 17948299

[B57] Medici D. Kalluri R. (2012). Endothelial-mesenchymal transition and its contribution to the emergence of stem cell phenotype. Semin. Cancer Biol. 22, 379–384. 10.1016/j.semcancer.2012.04.004 22554794 PMC3422405

[B58] Menon V. Eberth J. F. Goodwin R. L. Potts J. D. (2015). Altered hemodynamics in the embryonic heart affects outflow valve development. J. Cardiovasc. Dev. Dis. 2:108. 10.3390/jcdd2020108 26878022 PMC4751060

[B59] Midgett M. Goenezen S. Rugonyi S. (2014). Blood flow dynamics reflect degree of outflow tract banding in Hamburger-Hamilton stage 18 chicken embryos. J. R. Soc. Interface 11:20140643. 10.1098/rsif.2014.0643 25165602 PMC4191090

[B60] Midgett M. Rugonyi S. (2014). Congenital heart malformations induced by hemodynamic altering surgical interventions. Front. Physiol. 5:287. 10.3389/fphys.2014.00287 25136319 PMC4117980

[B61] Midgett M. Thornburg K. L. Rugonyi S. (2017). Blood flow patterns underlie developmental heart defects. Am. J. Physiol. Heart Circ. Physiol.. 10.1152/ajpheart.00641.2016 [Epub ahead of print]. 28062416 PMC5402020

[B62] Misra C. Sachan N. McNally C. R. Koenig S. N. Nichols H. A. Guggilam A.. (2012). Congenital heart disease–causing gata4 mutation displays functional deficits *in vivo*. PLoS Genet. 8:e1002690. 10.1371/journal.pgen.1002690 22589735 PMC3349729

[B63] Moonen J. R. Lee E. S. Schmidt M. Maleszewska M. Koerts J. A. Brouwer L. A.. (2015). Endothelial-to-mesenchymal transition contributes to fibro-proliferative vascular disease and is modulated by fluid shear stress. Cardiovasc. Res. 108, 377–386. 10.1093/cvr/cvv175 26084310

[B64] Nakajima Y. Morishima M. Nakazawa M. Momma K. Nakamura H. (1997). Distribution of fibronectin, type I collagen, type IV collagen, and laminin in the cardiac jelly of the mouse embryonic heart with retinoic acid-induced complete transposition of the great arteries. Anat. Rec. 249, 478–485. 9415455 10.1002/(SICI)1097-0185(199712)249:4<478::AID-AR7>3.0.CO;2-N

[B65] Nakajima Y. Yamagishi T. Hokari S. Nakamura H. (2000). Mechanisms involved in valvuloseptal endocardial cushion formation in early cardiogenesis: roles of transforming growth factor (TGF)-beta and bone morphogenetic protein (BMP). Anat. Rec. 258, 119–127. 10.1002/(SICI)1097-0185(20000201)258:2<119::AID-AR1>3.0.CO;2-U 10645959

[B66] Nimura K. Kaneda Y. (2016). Elucidating the mechanisms of transcription regulation during heart development by next-generation sequencing. J. Hum. Genet. 61, 5–12. 10.1038/jhg.2015.84 26202577

[B67] Norris R. A. Kern C. B. Wessels A. Moralez E. I. Markwald R. R. Mjaatvedt C. H. (2004). Identification and detection of the periostin gene in cardiac development. Anat. Rec. A Discov. Mol. Cell. Evol. Biol. 281, 1227–1233. 10.1002/ar.a.20135 15532025

[B68] Person A. D. Klewer S. E. Runyan R. B. (2005). Cell biology of cardiac cushion development. Int. Rev. Cytol. 243, 287–335. 10.1016/s0074-7696(05)43005-3 15797462

[B69] Reiner J. E. Datta P. K. (2011). TGF-beta-dependent and -independent roles of STRAP in cancer. Front. Biosci. 16, 105–115. 10.2741/3678 21196161 PMC4281466

[B70] Rennie M. Y. Gahan C. G. Lopez C. S. Thornburg K. L. Rugonyi S. (2014). 3D imaging of the early embryonic chicken heart with focused ion beam scanning electron microscopy. Microsci. Microanal. 20, 1111–1119. 10.1017/s1431927614000828 24742339 PMC4349375

[B71] Robinson M. D. McCarthy D. J. Smyth G. K. (2010). edgeR: a Bioconductor package for differential expression analysis of digital gene expression data. Bioinformatics 26, 139–140. 10.1093/bioinformatics/btp616 19910308 PMC2796818

[B72] Rugonyi S. Shaut C. Liu A. Thornburg K. Wang R. K. (2008). Changes in wall motion and blood flow in the outflow tract of chick embryonic hearts observed with optical coherence tomography after outflow tract banding and vitelline-vein ligation. Phys. Med. Biol. 53, 5077–5091. 10.1088/0031-9155/53/18/015 18723935

[B73] Runyan R. B. Heimark R. L. Camenisch T. D. Klewer S. E. (2000). Epithelial-mesenchymal transformation in the embryonic heart, in Madame Curie Bioscience Database [Internet] (Austin, TX: Landes Bioscience), 2000–2013.

[B74] Runyan R. B. Markwald R. R. (1983). Invasion of mesenchyme into three-dimensional collagen gels: a regional and temporal analysis of interaction in embryonic heart tissue. Dev. Biol. 95, 108–114. 6825921 10.1016/0012-1606(83)90010-6

[B75] Russo C.A. Elixhauser A. (2006). Hospitalizations for birth defects, 2004: statistical brief #24, in Healthcare Cost and Utilization Project (HCUP) Statistical Briefs (Rockville, MD: Agency for Healthcare Research and Quality US). 21938840

[B76] Samsa L. A. Givens C. Tzima E. Stainier D. Y. Qian L. Liu J. (2015). Cardiac contraction activates endocardial Notch signaling to modulate chamber maturation in zebrafish. Development 142, 4080–4091. 10.1242/dev.125724 26628092 PMC4712836

[B77] Sarkozy A. Conti E. Seripa D. Digilio M. C. Grifone N. Tandoi C.. (2003). Correlation between PTPN11 gene mutations and congenital heart defects in Noonan and LEOPARD syndromes. J. Med. Genet. 40, 704–708. 10.1136/jmg.40.9.704 12960218 PMC1735592

[B78] Schroeder J. A. Jackson L. F. Lee D. C. Camenisch T. D. (2003). Form and function of developing heart valves: coordination by extracellular matrix and growth factor signaling. J. Mol. Med. 81, 392–403. 10.1007/s00109-003-0456-5 12827270

[B79] Sedmera D. Pexieder T. Rychterova V. Hu N. Clark E. B. (1999). Remodeling of chick embryonic ventricular myoarchitecture under experimentally changed loading conditions. Anat. Rec. 254, 238–252. 9972809 10.1002/(SICI)1097-0185(19990201)254:2<238::AID-AR10>3.0.CO;2-V

[B80] Sewell-Loftin M. K. DeLaughter D. M. Peacock J. R. Brown C. B. Baldwin H. S. Barnett J. V.. (2014). Myocardial contraction and hyaluronic acid mechanotransduction in epithelial-to-mesenchymal transformation of endocardial cells. Biomaterials 35, 2809–2815. 10.1016/j.biomaterials.2013.12.051 24433835 PMC3950274

[B81] Shao D. D. Xue W. Krall E. B. Bhutkar A. Piccioni F. Wang X.. (2014). KRAS and YAP1 converge to regulate EMT and tumor survival. Cell 158, 171–184. 10.1016/j.cell.2014.06.004 24954536 PMC4110062

[B82] Shi L. Goenezen S. Haller S. Hinds M. T. Thornburg K. L. Rugonyi S. (2013). Alterations in pulse wave propagation reflect the degree of outflow tract banding in HH18 chicken embryos. Am. J. Physiol. Heart Circ. Physiol. 305, H386–H396. 10.1152/ajpheart.00100.2013 23709601 PMC3742871

[B83] Tobita K. Schroder E. A. Tinney J. P. Garrison J. B. Keller B. B. (2002). Regional passive ventricular stress-strain relations during development of altered loads in chick embryo. Am. J. Physiol. Heart Circ. Physiol. 282, H2386–H2396. 10.1152/ajpheart.00879.2001 12003850

[B84] Tomita-Mitchell A. Maslen C. L. Morris C. D. Garg V. Goldmuntz E. (2007). GATA4 sequence variants in patients with congenital heart disease. J. Med. Genet. 44, 779–783. 10.1136/jmg.2007.052183 18055909 PMC2652815

[B85] Vijayaraj P. Le Bras A. Mitchell N. Kondo M. Juliao S. Wasserman M.. (2012). Erg is a crucial regulator of endocardial-mesenchymal transformation during cardiac valve morphogenesis. Development 139, 3973–3985. 10.1242/dev.081596 22932696 PMC3472597

[B86] Wang Z. Calpe B. Zerdani J. Lee Y. Oh J. Bae H.. (2016). High-throughput investigation of endothelial-to-mesenchymal transformation (EndMT) with combinatorial cellular microarrays. Biotechnol. Bioeng. 113, 1403–1412. 10.1002/bit.25905 26666585

[B87] Webb S. Qayyum S. R. Anderson R. H. Lamers W. H. Richardson M. K. (2003). Septation and separation within the outflow tract of the developing heart. J. Anat. 202, 327–342. 10.1046/j.1469-7580.2003.00168.x 12739611 PMC1571094

[B88] Weismann C. G. Hager A. Kaemmerer H. Maslen C. L. Morris C. D. Schranz D.. (2005). PTPN11 mutations play a minor role in isolated congenital heart disease. Am. J. Med. Genet. A 136A, 146–151. 10.1002/ajmg.a.30789 15940693

[B89] Xu J. Lamouille S. Derynck R. (2009). TGF-beta-induced epithelial to mesenchymal transition. Cell Res. 19, 156–172. 10.1038/cr.2009.5 19153598 PMC4720263

[B90] Yang Q. Chen H. Correa A. Devine O. Mathews T. J. Honein M. A. (2006). Racial differences in infant mortality attributable to birth defects in the United States, 1989–2002. Birth Defects Res. A 76, 706–713. 10.1002/bdra.20308 17022030

[B91] Yu W. Huang C. Wang Q. Huang T. Ding Y. Ma C.. (2014). MEF2 transcription factors promotes EMT and invasiveness of hepatocellular carcinoma through TGF-beta1 autoregulation circuitry. Tumour Biol. 35, 10943–10951. 10.1007/s13277-014-2403-1 25087096

[B92] Zeisberg M. Neilson E. G. (2009). Biomarkers for epithelial-mesenchymal transitions. J. Clin. Invest. 119, 1429–1437. 10.1172/JCI36183 19487819 PMC2689132

[B93] Zhang H. von Gise A. Liu Q. Hu T. Tian X. He L.. (2014). Yap1 is required for endothelial to mesenchymal transition of the atrioventricular cushion. J. Biol. Chem. 289, 18681–18692. 10.1074/jbc.M114.554584 24831012 PMC4081914

[B94] Zhang W. Liu Y. Hu H. Huang H. Bao Z. Yang P.. (2015). ALDH1A3: A marker of mesenchymal phenotype in gliomas associated with cell invasion. PLoS ONE 10:e0142856. 10.1371/journal.pone.0142856 26575197 PMC4648511

[B95] Zhao D. Besser A. H. Wander S. A. Sun J. Zhou W. (2015). Cytoplasmic p27 promotes epithelial-mesenchymal transition and tumor metastasis via STAT3-mediated Twist1 upregulation. 34, 5447–5459. 10.1038/onc.2014.473 25684140 PMC4537852

